# The influence of different crosslinking agents onto the physical properties, integration behavior and immune response of collagen-based barrier membranes

**DOI:** 10.3389/fbioe.2024.1506433

**Published:** 2025-01-06

**Authors:** Yanru Ren, Said Alkildani, Kim Burckhardt, Alexander Köwitsch, Milena Radenkovic, Sanja Stojanovic, Stevo Najman, Ole Jung, Luo Liu, Mike Barbeck

**Affiliations:** ^1^ Clinic and Policlinic for Dermatology and Venereology, University Medical Center Rostock, Rostock, Germany; ^2^ BerlinAnalytix GmbH, Berlin, Germany; ^3^ Biotrics bioimplants AG, Berlin, Germany; ^4^ Department for Cell and Tissue Engineering, Faculty of Medicine, University of Niš, Niš, Serbia; ^5^ Department for Cell and Tissue Engineering, Scientific Research Center for Biomedicine, Faculty of Medicine, University of Niš, Niš, Serbia; ^6^ Department of Biology and Human Genetics, Faculty of Medicine, University of Niš, Niš, Serbia; ^7^ Beijing Advanced Innovation Center for Soft Matter Science and Engineering, College of Life Science and Technology, Beijing University of Chemical Technology, Beijing, China

**Keywords:** guided bone regeneration (GBR), collagen membrane, barrier membrane, transmembraneous vascularization, macrophages, degradation, integration

## Abstract

This study investigates the mechanical properties as well as *in vitro* and *in vivo* cyto- and biocompatibility of collagen membranes cross-linked with glutaraldehyde (GA), proanthocyanidins (PC), hexamethylendiisocyanate (HMDI) and 1-Ethyl-3-(3-dimethylaminopropyl) carbodiimide/N-hydroxysuccinimide (EC/NHS). A non-crosslinked membrane was used as reference control (RF). The initial *in vitro* cytotoxic analyses revealed that the PC, EC, and HMDI crosslinked membranes were cytocompatible, while the GA crosslinked membrane was cytotoxic and thus selected as positive control in the further *in vivo* study. Cross-linking enhances the tensile strength and collagenase resistance, effectively prolonging the membrane’s standing time *in vivo*. Using (immune-) histochemistry and histomorphometrical analyses, the cellular inflammatory responses, tissue integration and vascularization patterns at 10-, 30-, and 90-day post-implantation in a subcutaneous implantation model in rats were analyzed. The PC membrane elicited the mildest inflammatory cell levels, akin to the RF membrane, while other groups induced an M1-dominated macrophage response and numerous multinucleated giant cells throughout the study period. EC membranes maintained structural stability up to 30 days post-implantation, similar to the GA group, whereas others collapsed prematurely. Concurrent with membrane collapse, transmembrane vascularization occurred across all groups. Histopathological and histomorphometry results reveal the intricate interplay of inflammatory cell populations in vascularization. These findings offer valuable insights into the pivotal role of cross-linkers in modulating mechanical properties and tissue responses of collagen membranes.

## 1 Introduction

Over the past few years, barrier membranes have gained increasing prominence in the field of Guided Tissue Regeneration (GTR) and Guided Bone Regeneration (GBR) for the treatment of various defects such as horizontal and augmentation, ridge preservation and intraosseous defects. The first task for the application of a barrier membrane is the separation of the soft tissue and the bone defect area to prevent the migration of connective tissue into the intended regeneration site. Nowadays, more specific requirements for the “ideal barrier membrane” have been proposed to trigger clinical effectiveness including space maintenance, cellular occlusion, easy handling, and especially bioactive properties such as the transmembraneous vascularization or the induction of an (inflammatory) micromilieu that optimally supports bone tissue regeneration (IA et al., 2018; Omar et al., 2019; Sasaki et al., 2021).

As the most prominent structural protein of the extracellular matrix, collagen has earned its status as a very appropriate candidate for medical biomaterials for a broad variety of indications due to its exceptional biocompatibility, biodegradability, low immunogenicity, and cellular affinity (Chattopadhyay and Raines, 2014; Silvipriya et al., 2015; Pawelec et al., 2016). Collagen membranes have found widespread clinical use, expediting early wound stabilization and defect closure (Bunyaratavej and Wang, 2001; Allan et al., 2021). Additionally, their suitability for clinical procedures for GBR/GTR applications is underscored by the advantages of single-step application and low exposure rates (Gueldenpfennig et al., 2020; Kumari et al., 2021).

It is generally accepted that the barrier functionality should endure for 4–6 weeks in periodontal tissue regeneration and 16–24 weeks in case of bone augmentation procedures (Caballé-Serrano et al., 2019; Aprile et al., 2020; Sasaki et al., 2021). However, collagen-based materials especially derived from mammalian skin, which are most frequently used for production of barrier membranes, experience rapid degradation within a few days up to some weeks post-implantation (Sheikh et al., 2017). Multiple strategies have been introduced to increase the durability and mechanical strength of collagen membranes within the tissue, including alterations in membrane structure and collagen source (Schlegel et al., 1997; Wang et al., 2016; Noble et al., 2022). One of the most renowned commercial collagen membranes, Bio-Gide^®^, features a bilayer structure, with the dense layer remaining intact for up to 2 months *in vivo* (Schlegel et al., 1997; Radenković et al., 2021). Another clinically utilized membrane, Jason^®^, derived from porcine pericardium, boasts remarkable multidirectional tear resistance and sustained barrier function lasting 8–12 weeks (Fujioka-Kobayashi et al., 2017; Ratiu et al., 2019).

Among these strategies, cross-linking has gained significant attention for its effectiveness in enhancing the physicochemical and biological properties of collagen membranes by introducing inter- and intramolecular covalent or non-covalent bonds (Jiménez Garcia et al., 2017; Adamiak and Sionkowska, 2020). While glutaraldehyde (GA) is a highly efficient traditional chemical crosslinker for collagen-based materials, concerns have been raised regarding its propensity to induce localized cytotoxicity and significant inflammation (Ruijgrok et al., 1994; Nashchekina et al., 2020; Shi et al., 2020). Hexamethylendiisocyanate (HMDI) initially appeared as a promising substitute to GA, which also forms stable urea groups with the primary amine groups on collagen without toxic by-products (Olde Damink et al., 1995; Jarman-Smith et al., 2004; Sarrigiannidis et al., 2021). Notably, the commercially available collagen repair patch from Zimmer employs HMDI crosslinking, demonstrating a prolonged degradation period without triggering an increase in fibrinogen levels as an indicative of inflammation (Nicholson et al., 2007). In pursuit of safer alternatives, 1-Ethyl-3-(3-dimethylaminopropyl) carbodiimide/N-hydroxysuccinimide (EC-NHS) gained prominence as a zero-length cross-linking agent (Yang, 2012; Zhang et al., 2022). Applications of EC-NHS crosslinked collagen are diverse, and it has been proven that EC-NHS crosslinked collagen membrane induced successful bone regeneration in the Beagle mandible model and the rabbit calvaria defect model (Ahn et al., 2020). Moreover, in a study by Yang *et al.*, it was demonstrated that an increase of the EC concentration leads to a reduced swelling ratio and an enhancement in resistance to enzymatic degradation within collagen hydrogels (Yang, 2012). In addition to these advancements, proanthocyanidins (PC), a natural polyphenolic crosslinking agent, is generally considered to provide distinct advantages in terms of biocompatibility when compared to conventional chemical crosslinkers (Green et al., 2010; He et al., 2011). Moreover, PC exhibits a wide array of beneficial biological activities in the context of tissue regeneration including anti-bacterial, antioxidant, anti-inflammatory, and anti-tumor characteristics (Green et al., 2010; He et al., 2011; Rauf et al., 2019).

Although various cross-linking technologies for collagen implants and barrier membranes have been investigated, concerns have arisen due to crosslinker-induced foreign body reactions (Oryan et al., 2018; Adamiak and Sionkowska, 2020). The primary emphasis is consistently on enhancing the degradation pattern of a collagen membrane while minimizing foreign body reactions induced by crosslinkers. It is worth noting that the type and concentration of the crosslinker can trigger distinct immune responses and degradation patterns (Oryan et al., 2018; Sarrigiannidis et al., 2021). In this context, the comparison of the characteristic and composition of the foreign body reactions induced by different cross-linking agents is a pivotal endeavor in the evaluation of suitable cross-linking technologies.

Thus, novel bilayer collagen membranes manufactured through lyophilization and crosslinking with the four different cross-linking agents including GA, EC/NHS, PC, and HMDI were analyzed in the present study. Initially, the mechanical properties of the differently crosslinked membranes were compared by testing tensile strength, denaturation temperature, swelling rate, and collagenase resistance. Furthermore, an *in vitro* cytotoxicity part followed by an *in vivo* study part including analyses of the tissue integration, angiogenesis and immune pattern using the by subcutaneous implantation model in rats up to 90 days were explored. Established and previously published methodologies especially focused on the histological and histomorphometrical analyses were used (Kapogianni et al., 2021; Radenković et al., 2021; Alkildani et al., 2023). By comparing *ex vivo*, *in vitro* and *in vivo* characteristics exhibited by the differently crosslinked collagen membranes, this study aimed to provide valuable insights for the further development of clinically optimal collagen membranes.

## 2 Methods and materials

### 2.1 Preparation of dual-layer collagen membranes

**FIGURE 1:**
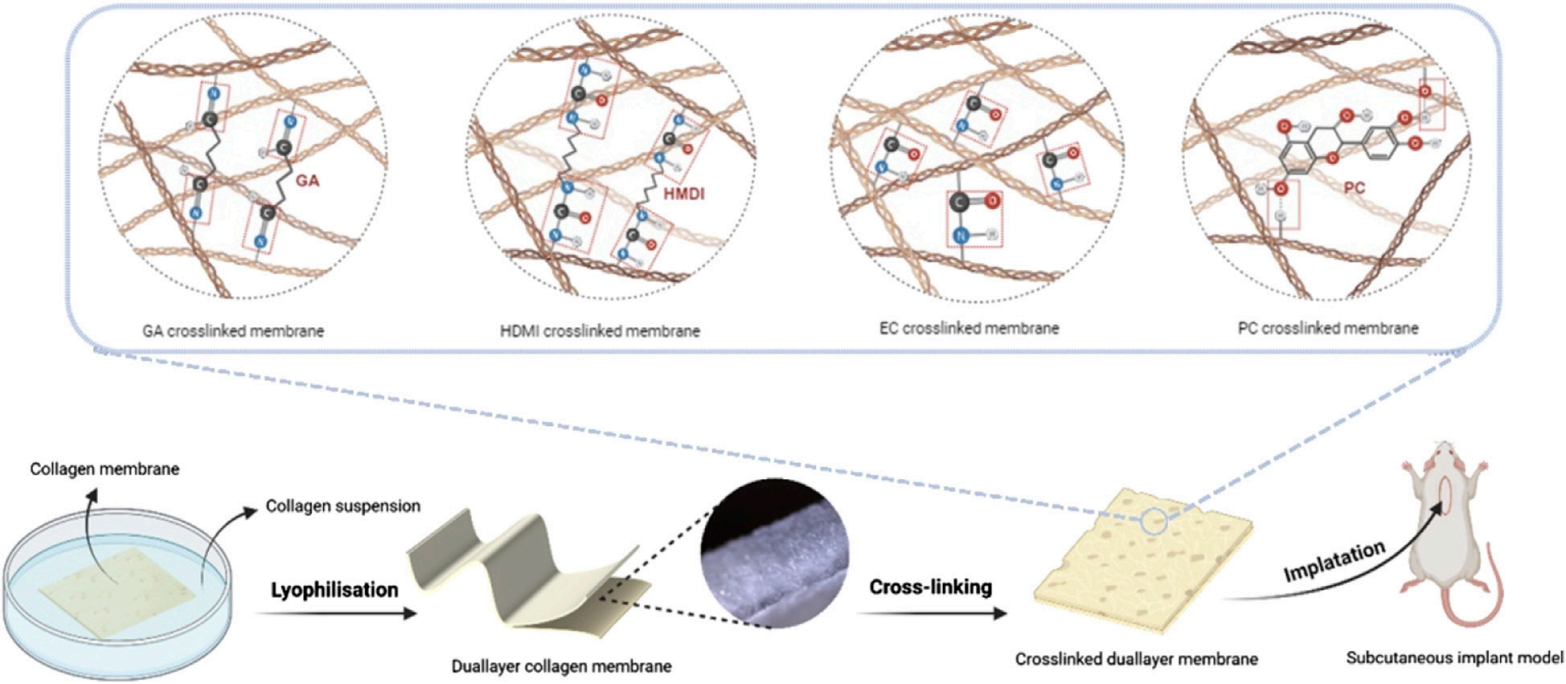
Schematic illustration of the preparation processes.

A native collagen membrane sourced from porcine dermis (Collprotect, botiss biomaterials GmbH, Zossen, Germany) served as the base layer (BL) and was initially cut into 5 × 5 cm dimensions for the subsequent steps. Additionally, porcine skin was crushed and subsequently homogenized in trisodium phosphate buffer utilizing an IKA Ultra-Turrax T-25 Digital Homogenizer. The collagen extracted from this process was then re-diluted with water to create a 0.75% collagen suspension at a pH of 7. The collagen suspension was poured into a mold, and the base layer was placed on top of the suspension. The final dullayer membrane, consisting of a fleece layer made from the collagen suspension and a base layer, was then formed through freeze-drying (Figure 1).

### 2.2 Crosslinking processes

For the present study, four different cross-linking agents, i.e., proanthocyanidin (PC), glutaraldehyde (GA), 1-Ethyl-3-(3-dimethylaminopropyl) carbodiimide (EC) and hexamthylene diisocyanato (HMDI) in two different concentrations (H1 and H2) were used for the preparation of new collagen membrane prototypes (Table 1). A dual-layer membrane without further cross-linking was used as reference membrane (RF) or negative control group (Table 1).

**TABLE 1:** Cross-linking details of the collagen membranes.

Group	Cross-link agent	Amount of collagen	Amount of crosslinker	Concentration
RF	-	-	-	-
PC	Proanthocyanidin	1,496 mg	1,496 mg	100 wt%/g collagen
EC	1-Ethyl-3-(3-dimethylaminopropyl) carbodiimide	1,633 mg	EC 1878.28 g NHS 451 g	6 mmol/g collagen
H1	Hexamthylene diisocyanat	1,616 mg	808 mg	50 wt%/g collagen
H2	Hexamthylendiisocyanat	1,443 mg	1,443 mg	100 wt%/g collagen
GA	Glutaraldehyde	1,617 mg	2021 mg	125 wt%/g collagen

#### 2.2.1 EC/NHS crosslinking

To produce the EC/NHS crosslinked membrane, the fleece layer of the bilayer membrane obtained in the previous step was initially soaked in 95% isopropanol (Geyer Chemsolute, Renningen, Germany) for 10 min. EC (Carl Roth, Karlsruhe, Germany) and NHS (Carl Roth, Karlsruhe, Germany) were separately weighed in a molar ratio of 2:1. Each compound was dissolved in 40 mL of 95% isopropanol and then introduced into the vessel containing the bilayer membranes. The reaction proceeded for 24 h. Following this, the EC crosslinked membrane underwent two washing steps with 95% isopropanol for 2 min each using an ultrasonic bath. It was subsequently washed once with 95% isopropanol for 10 min and once with 100% isopropanol for 5 min under stirring. The final membrane was obtained after air drying with lint-free cloths (KIMTECH SCIENCE* Precision Wipes, Kimberly-Clark, Roswell, GA, United States) in a fume hood.

#### 2.2.2 PC crosslinking

To create the PC crosslinked membrane, PC (Grape seed extract pure, PureBulk, Inc., Roseburg, U.S.) was accurately weighed and introduced into 300 mL of 90% isopropanol. Subsequently, the fleece layer of the membrane was immersed in the PC solution for a duration of 24 h. Following this soaking period, the membrane underwent two successive washes with 90% isopropanol for 10 min each, followed by a final wash with 100% isopropanol under continuous stirring. The ultimate membrane product was obtained after air drying using lint-free cloths (KIMTECH SCIENCE* Precision Wipes, Kimberly-Clark, Roswell, GA, United States) within a fume hood.

#### 2.2.3 HMDI crosslinking

To prepare the HMDI crosslinked membrane layer, a 40 mg/mL HMDI (Sigma-Aldrich, St. Louis, MO, U.S.) solution in 95% isopropanol was initially prepared. Two HMDI crosslinked membranes, designated as H1 (50 wt%) and H2 (100 wt%), were then subjected to crosslinking in HMDI solutions with varying concentrations. To prevent the precipitating urea compound that inhibit the crosslinking reaction from coating on the collagen, the collagen membranes were immersed separately in 20.2 mL (for H1 membrane) and 36.1 mL (for H2 membrane) of 40 mg/mL HMDI solution for 5 min. Following this, the reaction solution was diluted to a total volume of 150 mL, and the reaction was initiated by adding NaOH solution (with a molar ratio of NaOH to HMDI as 1:25) to the fleece layer. The reaction was allowed to proceed for 24 h. Afterward, the HMDI crosslinked membranes underwent two washes with 100% isopropanol for 2 min each using an ultrasonic bath. They were then washed once with 100% isopropanol for 20 min under continuous stirring. The final membrane product was obtained after air drying using lint-free cloths (KIMTECH SCIENCE* Precision Wipes, Kimberly-Clark, Roswell, GA, United States) within a fume hood.

#### 2.2.4 GA crosslinking

To create the GA crosslinked membrane, GA (Thermo Fisher Scientific Inc., Waltham, Massachusetts, U.S.) was accurately weighed and dissolved in 140 mL of 95% isopropanol. This solution was then added to the fleece layer of the membrane for a 1-hour crosslinking process. Subsequently, the membrane was washed twice with 95% isopropanol for 2 min each, using an ultrasonic bath, and once with 100% isopropanol for 10 min under stirring. The final membrane was obtained after air drying using lint-free cloths (KIMTECH SCIENCE* Precision Wipes, Kimberly-Clark, Roswell, GA, United States) within a fume hood.

### 2.3 *Ex vivo* tests

#### 2.3.1 Collagenase assay

The stock solution of collagenase (Sigma Aldrich C9891) was diluted in TESCA buffer to achieve a concentration of 1 mg/mL (PH 7.4). The needed volume per sample tube is calculated under consideration of the enzyme activity given as collagen digestion unit (CDU). The collagen samples, with and without collagenase, were accurately weighed using a Kern ABT 120-5DNM balance and placed into individual Eppendorf tubes. These samples were then hydrated in TESCA buffer (excluding collagenase) for a duration of 30 min. The calculated volume of the collagenase solution was thoroughly mixed into each sample tube (1 CDU/mg collagen), and subsequently, the tubes were placed in a preheated incubator (Thermocycler, Thermomixer C, Eppendorf) at 37°C. At different timepoints, the digestion reaction was halted by centrifuging the sample tubes for 10 min at 4°C, utilizing an Eppendorf 5424 R centrifuge. Following centrifugation, the supernatant was meticulously decanted, and the samples were washed with deionized water, with each cycle involving centrifugation and removal of the supernatant. This washing process was repeated twice. Subsequently, the samples were left to air dry within a desiccator for 48 h before their residual collagen mass was weighed.

#### 2.3.2 Differential scanning calorimetry (DSC)

DSC measurements were conducted using a DSC 214 Polyma instrument (NETZSCH-Gerätebau GmbH, Germany). Initially, the samples were hydrated in deionized water and then transferred into Concavus Pans (Netzsch, NGB817525). Subsequently, they were weighed using an analytical balance (ABT 120-5DNM, Kern and Sohn GmbH) and sealed with Concavus Lids (Netzsch, NGB817526). The heating rate applied during the measurements was set at 10 K/min. Before performing the sample measurements, a correction and reference measurement were carried out. The obtained results were evaluated using the *Proteus* software (Netzsch).

#### 2.3.3 Tensile test

The tensile strength of the specimens was assessed using a tensile testing machine (RetrolineZ2.5, ZwickRoell GmbH and Co. KG). To prepare the samples, they were first cut to the required dimensions and then hydrated in deionized water for a duration of 2 min. Subsequently, the thickness of each sample was measured at three distinct points in the central region using a thickness measuring device (C110T, Kroeplin GmbH). The minimum recorded thickness value among the three measurements was utilized for calculating the tensile strength. The specimens were securely clamped, and the measurements were carried out with a 50 N load cell. The evaluation of the measurements was performed using the testXpert^®^ II software (ZwickRoell).

#### 2.3.4 Swelling ratio

The swelling ratio of a collagen membrane can be calculated by measuring the weight of the membrane before and after it absorbs liquid. After soaking for different times, the wet weight of membrane was recorded and then the swelling ratio was calculated according to the following equation:

$$Swelling\;ratio=\frac{W_{wet\;}-W_{dry}}{W_{dry}}\times 100\%$$

where: W_wet_ is the weight of the membrane after swelling (wet weight), W_dry_ is the weight of the membrane in its dry state.

### 2.4 In vitro experiments

#### 2.4.1 Cell culture and extraction

Cytocompatibility assessments were carried out in accordance with ISO 10993–5/12. Briefly, L-929 mouse fibroblast cells, sourced from the European Collection of Cell Culture (ECACC) in Salisbury, UK, were cultured in standard cell culture conditions with appropriate cell culture medium. When the cell culture reached approximately 80% confluency, cells were passaged.

All test samples were extracted after a 72-hour cultural period under standard cell culture conditions, maintaining a surface-to-volume ratio of 3 cm^2^/mL in cell culture medium. As a control, an extraction was performed using cell culture medium alone. Afterwards, the extract medium underwent centrifugation at 14,000 rpm for 10 min. The supernatants were used for further L929 cells culture that described below.

Subsequently, 96-well plates were seeded with 1 × 10^4^ cells per well in 100 µL of cell culture medium and cultured for 24 h under standard cell culture conditions. Following this incubation period, the cell culture medium was aspirated, and 100 µL of the extract solutions were added to each well. After an additional 24-hour incubation, the cells were subjected to analysis using BrdU- and XTT-assays. Simultaneously, the supernatants underwent LDH assay. Besides, parallel assays were performed for all extracts, excluding cells, to serve as a control for potential assay interference. RM-A, a polyurethane film containing 0.1% zinc diethyldithiocarbamate (ZDEC), obtained from the Hatano Research Institute, Food and Drug Safety Center in Japan, was utilied as the positive control. Absorbance values obtained from blank controls (comprising medium without cells) were subtracted from the results of all assays.

#### 2.4.2 Sodium 3,3′-[1(phenylamino)carbonyl]-3,4-tetrazolium]-3is (4-methoxy-6-nitro) Benzene Sulfonic acid Hydrate (XTT) assay

The Cell Proliferation Kit II from Roche Diagnostics in Mannheim, Germany, was employed following the manufacturer’s guidelines. In brief, the electron-coupling reagent was mixed with the XTT labeling reagent at a 1:50 dilution, and 50 μL of this mixture was added to the cells. Following a 4-hour incubation period under cell culture conditions, the conversion of the substrate was assessed by measuring the absorbance of 100 μL aliquots in a new 96-well plate using a scanning multi-well spectrophotometer (ELISA reader) equipped with filters for 450 nm and 650 nm (reference wavelength).

#### 2.4.3 Bromodeoxyuridine (BrdU) assay

The BrdU (colorimetric) test kit from Roche Diagnostics in Mannheim, Germany, was employed following the manufacturer’s guidelines. Briefly, cells were incubated with BrdU for 2 h under cell culture conditions, followed by fixation with FixDenat reagent for 30 min at room temperature. Subsequently, cells were incubated with an anti-BrdU peroxidase (POD) antibody for 1 h and then subjected to three 5-minute rinses with washing buffer. The addition of Tetramethyl-benzidine (TMB) to the substrate initiated a reaction, which was halted after 20 min at room temperature by adding 25 µL of 1 M H_2_SO_4_. Finally, the resulting immune complexes were quantified using a scanning multi-well spectrophotometer (ELISA reader) at wavelengths of 450 nm and 690 nm (reference wavelength).

#### 2.4.4 Lactate dehydrogenase (LDH) assay

The LDH Cytotoxicity Assay Kit II from BioVision in Milpitas, CA, United States, was employed following the manufacturer’s instructions. Specifically, 10 µL of cell supernatants were mixed with 100 µL of LDH reaction mix and incubated for 30 min at room temperature. Subsequently, stop solution was added, and the absorbance was measured using a scanning multi-well spectrophotometer (ELISA reader) at wavelengths of 450 nm and 650 nm (reference wavelength).

### 2.5 In vivo experiments

#### 2.5.1 Study design

The *in vivo* experiments were conducted in collaboration with the Faculty of Medicine at the University of Niš, Serbia. The animal study was approved by the Local Ethical Committee of the Faculty of Medicine, University of Niš and by the Veterinary Directorate of the Ministry of Agriculture, Forestry and Water Management of the Republic of Serbia (decision number 323–07-09101/2020–05/5; date of approval: 26 August 2020). A total of 90 male Wistar rats, aged 3–4 months, from the Vivarium of the Faculty of Medicine (University of Niš, Serbia) were divided into six study groups. Each group comprised 15 experimental animals, with five animals designated for each of the three time points (n = 5) at 10, 30, and 90 days in each group. These experimental animals were accommodated under standard conditions, which included access to water *ad libitum*, exposure to artificial lighting, and provision of regular rat pellets. Additionally, standard pre- and postoperative care procedures were diligently administered.

#### 2.5.2 Subcutaneous implantation and explantation procedure

The implantation procedure closely adhered to the protocol outlined by Barbeck and colleagues (Barbeck et al., 2014; Barbeck et al., 2016; Barbeck et al., 2020; Barbeck et al., 2022; Flaig et al., 2020). Briefly, the animals underwent anesthesia through intraperitoneal injection, which consisted of ketamine [100 mg/kg of body weight] and xylazine [5 mg/kg of body weight]. Following the administration of anesthesia and subsequent preparation, including shaving and disinfection, an incision was made extending down to the subcutaneous tissue within the rostral subscapular region. Subsequently, a subcutaneous pocket was gently created using scissors, and the biomaterials were placed within this pocket. Following the implantation, the incisions were sutured.

Afterwards, following the euthanasia of the animals using an overdose of a ketamine and xylazine mixture, the implanted membranes were removed at the different timepoints, directly fixed in 4% formalin for a duration of 24 h, and then sectioned into three equal segments. For tissue processing a series of dehydration steps using increasingly concentrated alcohol solutions and xylol were applied. Then, the samples were embedded in paraffin, and histological sections with a thickness ranging from 3 to 5 µm were prepared using a rotary microtome (Leica, Wetzlar, Germany).

#### 2.5.3 (Immuno-) histochemical staining

From each tissue block, four sections were sliced and subsequently utilized for (immuno-) histochemical staining procedures, which included hematoxylin and eosin (H&E) staining, CD163 and CD11c staining, as well as CD31 staining. The CD163 marker is specific to the anti-inflammatory M2 phenotype of macrophages, while the CD11c marker is specific to the pro-inflammatory M1 phenotype of macrophages. Moreover, CD31 serves as a common marker for identifying blood vessels, specifically marking endothelial cells. The execution of all staining protocols strictly adhered to previously published procedures (Flaig et al., 2020; Barbeck et al., 2022).

#### 2.5.4 Histo (patho)logical analyses

In this study, all histopathological and histomorphometrical analyses described in the following sections were conducted on the fleece layer of the dual-layer collagen membranes.

Initially, specimens of all membranes were histologically analyzed *ex vivo* to obtain more information about the material structure and further relations to the *in vivo* structure based by the cross-linking technologies as previously described (Barbeck et al., 2015; Kapogianni et al., 2021). To assess *in vivo* parameters such as cellular involvement in the integration and degradation processes of the tested membranes, as well as inflammatory tissue reactions, histopathological analyses were conducted using an Axio. Scope. A1 light microscope (Zeiss, Oberkochen, Germany). Histological images were captured with a connected Axiocam 305 color camera and processed using the ZEN Core software (Zeiss, Oberkochen, Germany).

#### 2.5.5 Histomorphometrical analysis

Utilizing ImageJ (Version 1.52t, U. S. National Institutes of Health, Bethesda, ML, United States of America) a histomorphometrical analysis was conducted on the immunohistochemically stained slides, which had initially been digitized using the PreciPoint M8 microscope (Precipoint GmbH, Munich, Germany). The objective was to derive data pertaining to macrophage subtypes and vascularization.

To quantify the presence of M1 and M2 macrophages, the adjacent soft tissue within the defect area was manually delineated. Subsequently, a specialized plugin, as described by Lindner *et al.*, was employed to automatically calculate the area occupied by stained cells within the delineated total area (Lindner et al., 2020). This procedure yielded the area of positive cells per square millimeter (in %). In addition, multinucleated giant cells (MNGCs) were counted manually.

To assess vascularization, a dedicated plugin also developed by Linder *et al.* was utilized to independently quantify the number and area of blood vessels within both the delineated membrane area and the peri-membrane area as previously described (Barbeck et al., 2015; Lindner et al., 2020). Subsequently, calculations were performed to obtain the vessel density (number of vessels per mm^2^) and vessel area fraction (%).

### 2.6 Statistical analyses

The data were presented as mean values along with standard deviations, utilizing GraphPad Prism software (Version 9.0.0, GraphPad Software Inc., La Jolla, United States of America). Afterwards, the statistical analysis of the data was conducted using two-way analysis of variance (ANOVA). Subsequently, a Tukey *post hoc* assessment was performed for group comparisons with the assistance of the GraphPad Prism software. Inter- and intraindividual significances were acknowledged when *p*-values were less than 0.05 (#/*/^·^ *p* < 0.05), denoting a significant difference. High significance was attributed to cases where the *p*-values were less than 0.01 (##/**/^..^ *p* < 0.01), 0.001 (###/***/^…^ *p* < 0.001) or less than 0.0001 (####/****/^.…^ *p* < 0.0001).

## 3 Results

### 3.1 Morphology of the prepared bilayer collagen membranes

**FIGURE 2:**
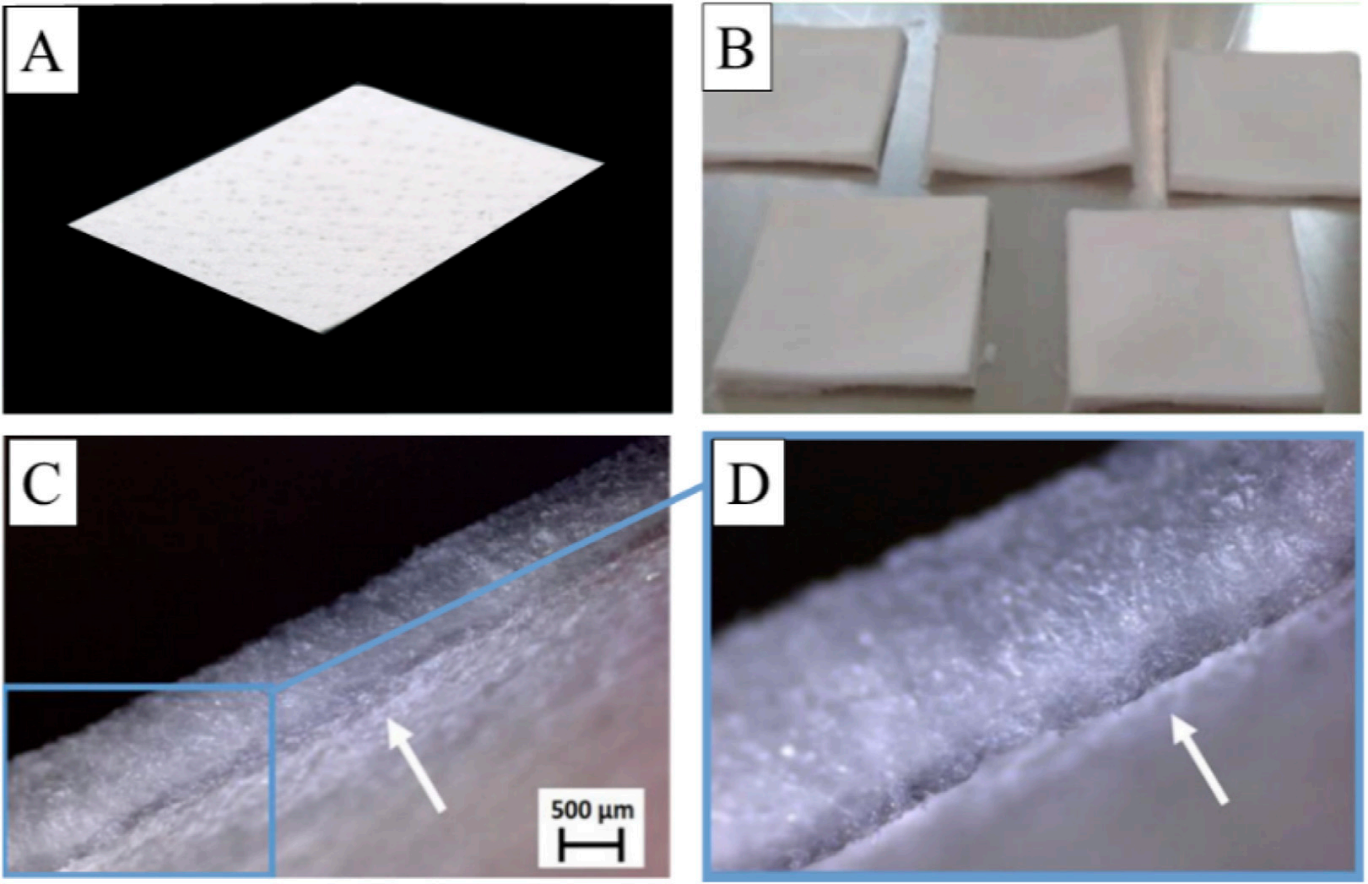
Macroscopic and microscopic morphology of the collagen membranes. **(A)** Native collagen membrane derived from porcine skin used as base layer; **(B)** prepared bilayer collagen membranes; **(C, D)** microscopic morphology of a reference membrane showing the non-cross-linked fleece layer on top of the native collagen membrane (white arrows).

The native collagen membrane with natural pores served as base layer (BL) during preparation of the bilayer collagen membrane. The base layer exhibits a dense and uniform structure with a thickness of approximately 0.4 mm (Figure 2A). Once a fleece layer was produced on top of the base layer, the thickness of the bilayer membrane increased significantly (Figure 2B). No visible pores were observed on the surface of the fleece layer, while its overall structure appears much fluffier compared to the base layer (Figures 2C, D). Thereby, no gap between the two layers was observable, indicating the formation of an irreversible interlayer connection (Figures 2C, D). In summary, a homogeneous and tightly connected bilayer collagen membrane was successfully created through the lyophilization process.

**FIGURE 3:**
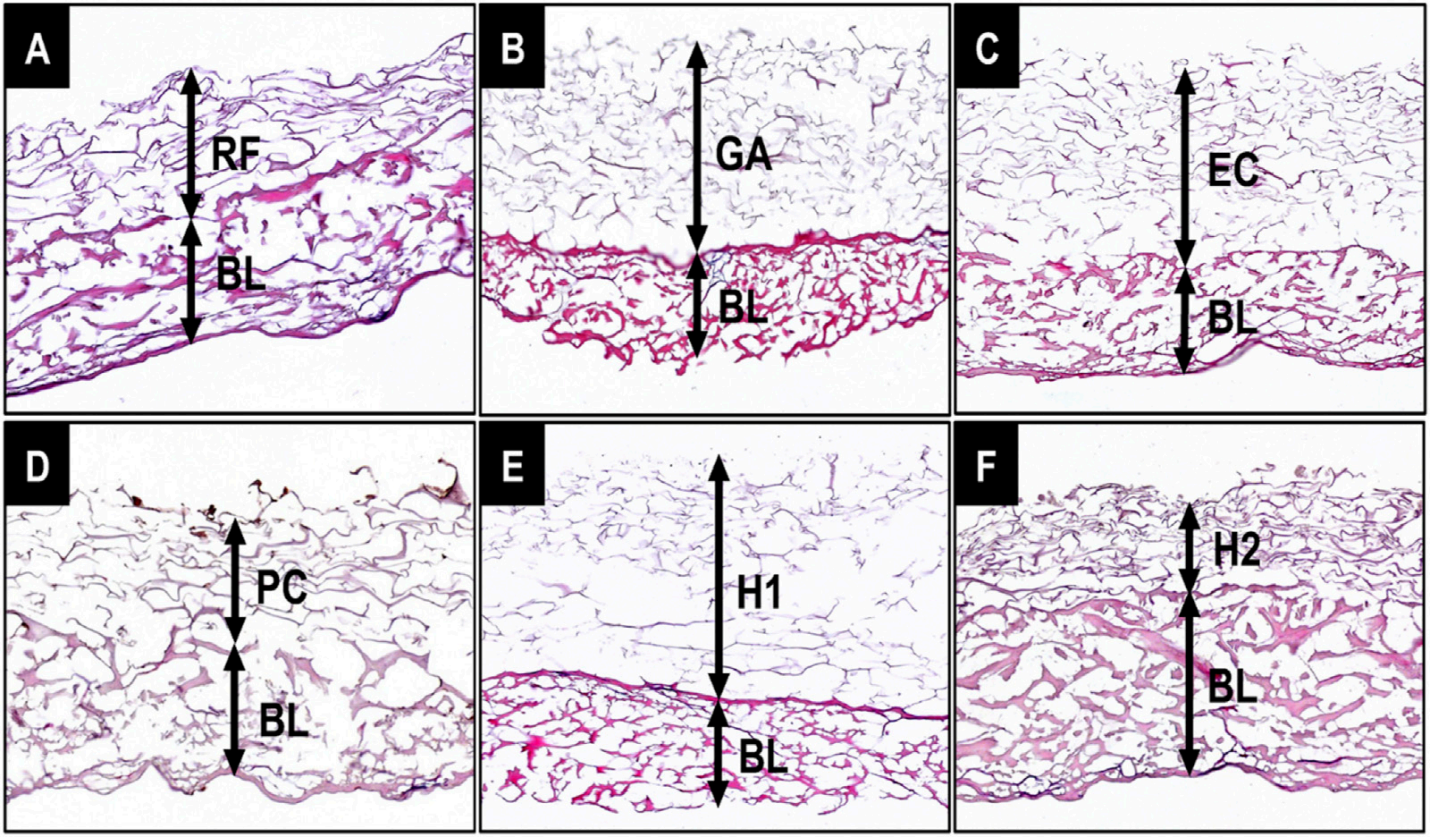
Histological visualization of the bi-layered barrier membranes consisting of the base layer (BL) and the fleece layers with and without cross-linking. **(A)** Reference membrane with the native non-crosslinked collagen fleece layer (RF); **(B)** Glutaraldehyde cross-linked fleece layer (GA); **(C)** EC/NHS cross-linked fleece layer (EC); **(D)** Proanthocyanidin cross-linked fleece layer (PC); **(E, F)** Hexamthylendiisocyanat cross-linked fleece layers (H1 and H2) (HE-stainings, excerpts of total scans, 100x magnifications).

The histological examination of the membranes showed that all membranes exhibited a dual-layer structure, comprising the base layer (BL), characterized by thick collagen fibers, and the upper fleece layer (RF, GA, EC, PC, H1, and H2), prepared from a collagen suspension, featuring thinner collagen fibers (Figure 3). In case of all materials, both layers displayed a honeycomb-like pore structure (Figure 3). Interestingly, the microstructure of the cross-linked membranes (Figures 3B–F) appeared to undergo minimal alteration compared to reference membrane (Figure 3A). Neither tissue nor cells were observed within these membranes.

### 3.2 Cytocompatibility analyses

**FIGURE 4:**
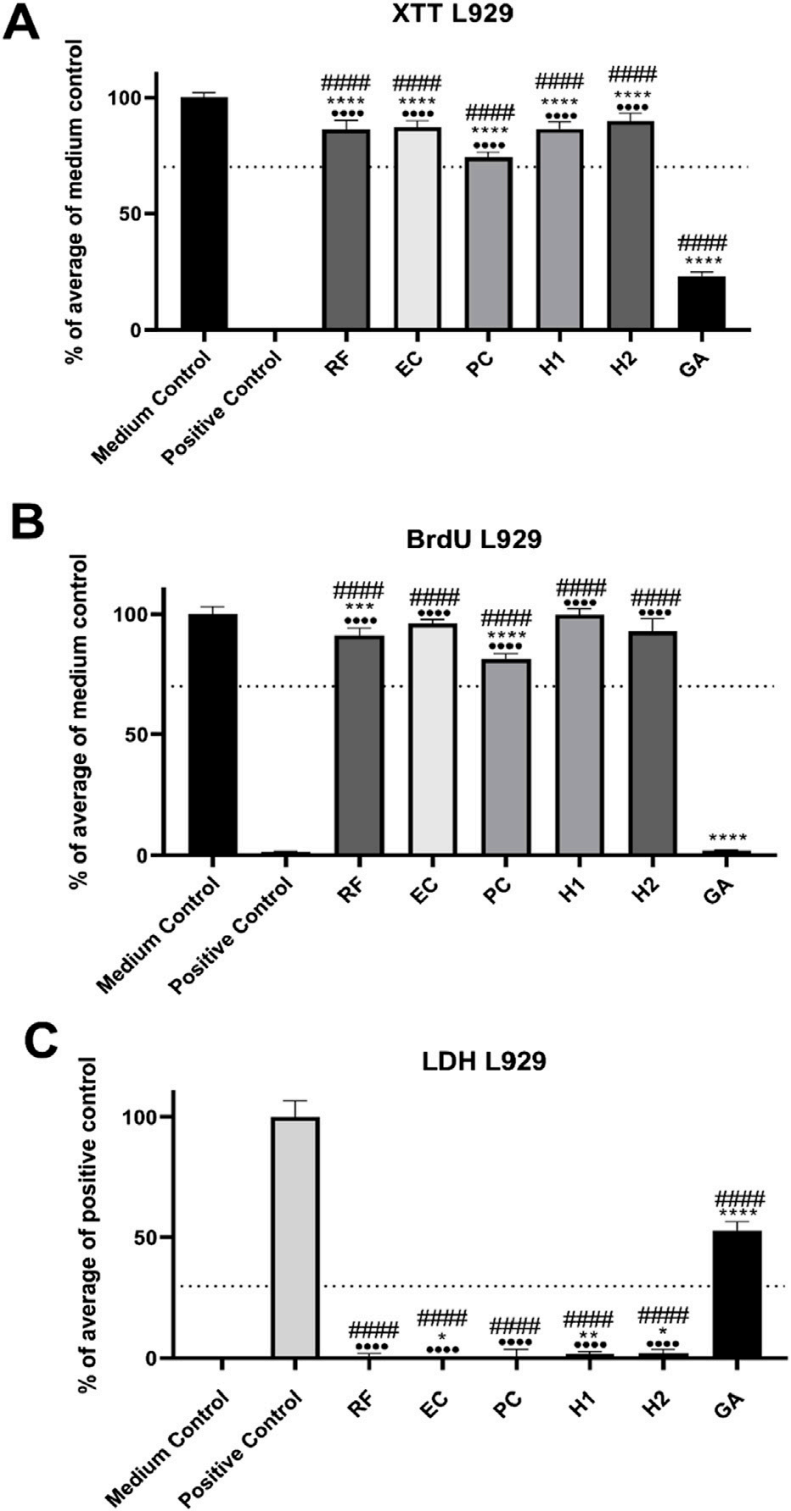
Results of the cytocompatibility analyses of the collagen membranes. **(A)** Assessment of viability via XTT assay; **(B)** Measurement of proliferation via BrdU assay; **(C)** Evaluation of cytotoxicity via LDH assay. Values are normalized against either positive controls (LDH) or a medium control (XTT, BrdU). The means are presented with error bars representing standard deviations. The dotted line signifies thresholds that should not be surpassed (LDH) or dropped below (XTT; BrdU). Statistical significance: */#*p* < 0.05, **/##*p* < 0.01, ***/###*p* < 0.001, ****/####/····*p* < 0.0001. * indicates comparison with medium control, # indicates comparison with positive control (RM-A), ·indicates comparison with GA group.

Three different *in vitro* assays were combined used to assess the cytotoxicity of the non-crosslinked reference and crosslinked membranes. RM-A film was used as positive control *in vitro* analysis. According to the ISO 10993–5 protocol, the sample with a L929 cell viability/proliferation above 70% of the medium control in XTT/BrdU assays, and a cytotoxicity below 30% of the medium control in LDH assays, were considered cytocompatible (Jung et al., 2019). Except for the GA group, the values in all membrane groups demonstrated good cytocompatibility in all three assays (Figure 4). Thus, all membrane types allowed for viability and proliferation above 70% (Figures 4A, B) combined with a cytotoxicity below 30% (Figure 4C).

However, the viability values in all membrane groups were significantly lower compared to the medium control (****p < 0.0001) but significantly higher compared to the values in the positive control group (####*p* < 0.0001). Finally, the values in all membrane groups were significantly higher compared to the values in the group of the GA membrane (^.…^ *p* < 0.0001) (Figure 4A).

The proliferation measurements showed that only the values in the groups of the reference membrane and the PC membrane differed significantly from that in the medium control group (***p < 0.001 and ****p < 0.0001), while the values in the groups of the EC membrane and both HMDI membranes were comparable (Figure 4B). Furthermore, the values in the GA group were significantly lower compared to that in the medium control group (****p < 0.0001). Moreover, the values in the afore mentioned membrane groups were significantly higher compared to the values in the positive control group (####*p* < 0.0001) and the group of the GA membrane (^.…^ *p* < 0.0001) (Figure 4B). No significances between the values in the positive control group and the GA group were measured.

The cytotoxicity measurement indicated that all membrane types induced significantly lower values compared to the values in the positive control group (####*p* < 0.0001) and the group of the GA membrane (^.…^ *p* < 0.0001) (Figure 4C). Thereby, the values in the RF and PC membrane groups were comparable to that in the medium control group, while the values in the groups of the EC membrane (*p < 0.05) and both HMDI membranes were significantly higher (*p < 0.05 and **p < 0.01) (Figure 4C).

### 3.3 Performance tests

To evaluate the mechanical properties of the crosslinked membranes, a series of tests including tensile testing, elongation at break, Differential Scanning Calorimetry (DSC), and swelling ratio were conducted.

**FIGURE 5:**
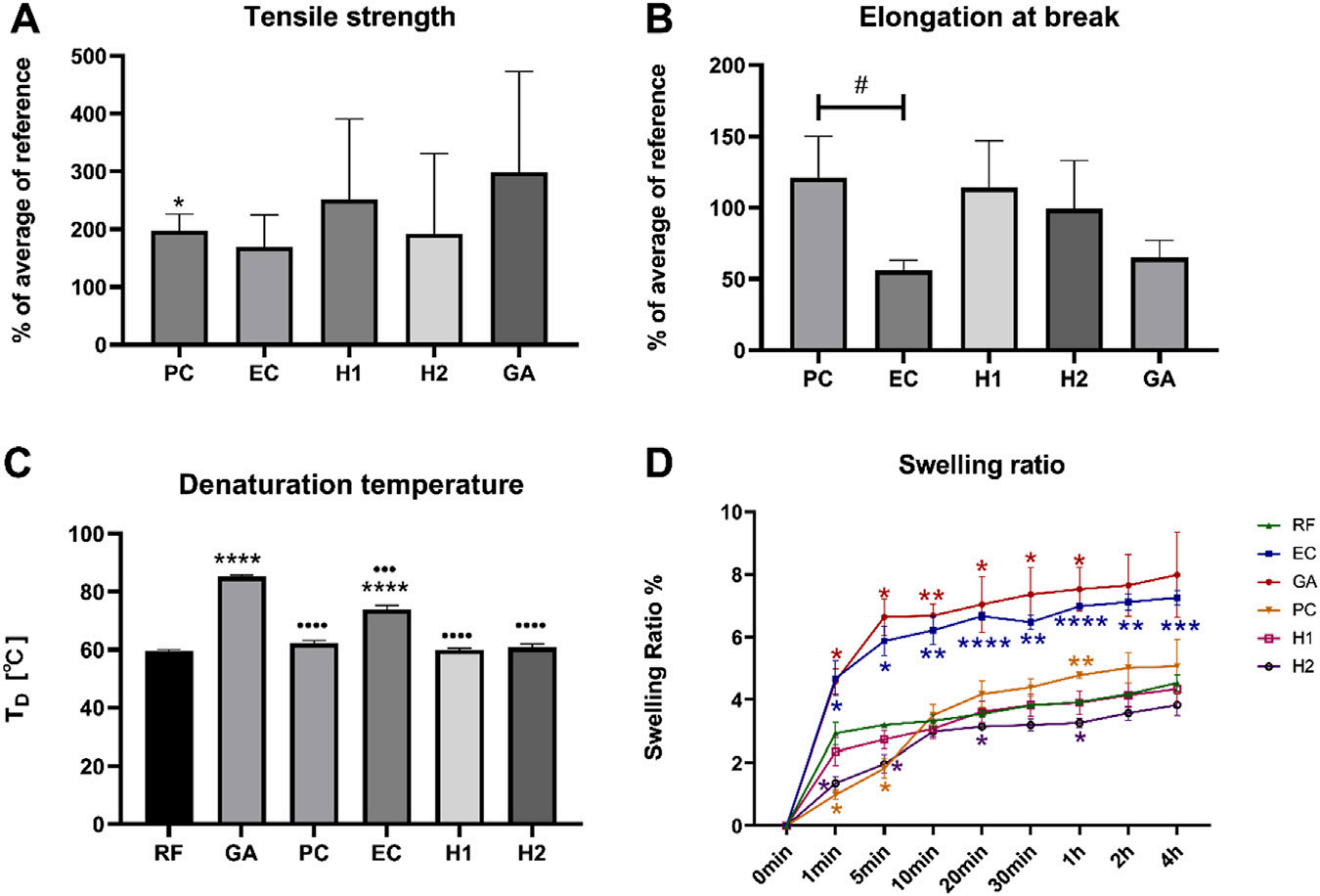
Results of the mechanical evaluation of crosslinked membranes. **(A)** Relative tensile strength; **(B)** elongation at break; **(C)** denaturation temperature (T_D_); **(D)** swelling ration. Statistical significance: */^.^/#*p* < 0.05, **p < 0.01, ***/^.…^ *p* < 0.001, ****/^.…^ *p* < 0.0001. * indicates comparison with RF, · indicates comparison with GA, # indicates interindividual significance.

The tensile strength tests showed that all crosslinked membranes exhibited increased mean tensile strength, but only PC membrane displayed a significant difference compared to the RF group (*p < 0.05) (Figure 5A). Additionally, the PC membrane also demonstrated higher elongation at break values compared to the EC membrane group (^#^ *p* < 0.05) (Figure 5B).

The DSC analysis measuring the denaturation temperature of the tested membranes showed that all membranes exhibited an increase in denaturation temperature following crosslinking (Figure 5C). Remarkably, the denaturation temperature of the GA membrane showed the most significant improvement with a denaturation temperature significantly higher than that of all the other membranes (^…^ *p* < 0.001, ^.…^ *p* < 0.0001). Furthermore, the EC membrane also displayed a significantly enhanced denaturation temperature compared to the RF membrane (****p < 0.0001).

The results of the swelling ratio test in Simulated Body Fluid (SBF) buffer showed that all membranes initially underwent rapid swelling within the first 5 min (Figure 5D). Afterwards, the swelling rate slowed down in all groups reaching a steady state at 20 min. In comparison to the RF membrane, the EC and GA membranes exhibited a faster swelling rate and higher swelling ratio (*p < 0.05, **p < 0.01, ***p < 0.001, ****p < 0.0001). The PC membrane displayed the lowest swelling rate in the initial stage (*p < 0.05), but it ultimately exhibited a higher swelling ratio than the RF membrane in the later stage (**p < 0.01). The H1 membrane exhibited a swelling pattern most similar to the RF membrane, whereas the H2 membrane, featuring a higher crosslinker concentration, displayed the lowest swelling rate and swelling ratio (*p < 0.05).

### 3.4 Collagenase assay

**FIGURE 6:**
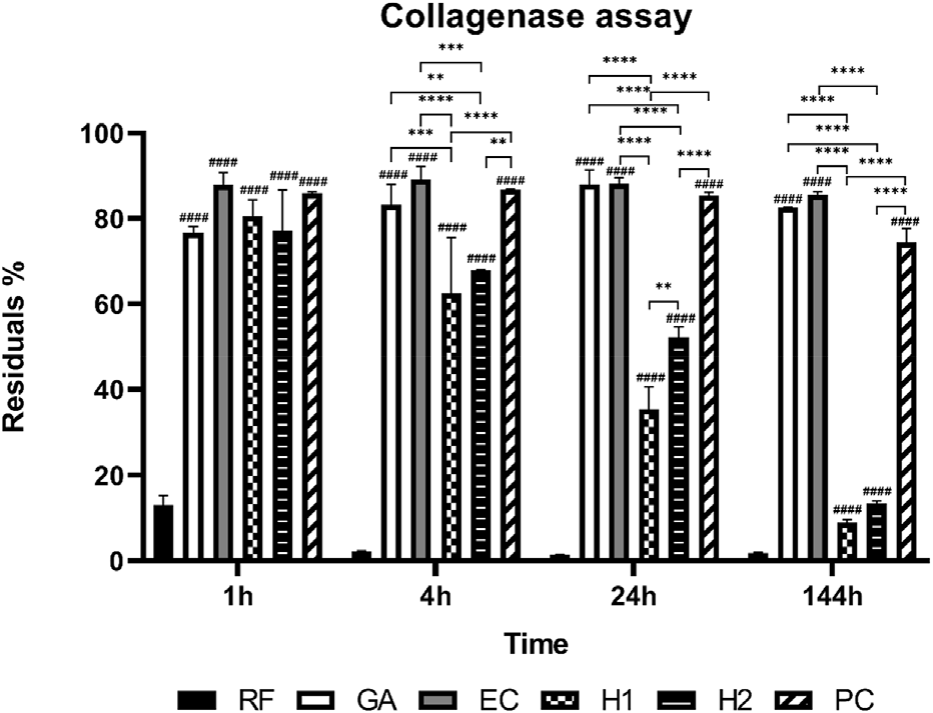
Degradation behavior of the cross-linked membranes (n = 3). Statistical significance: **p < 0.05, ***p < 0.001, ****/####*p* < 0.0001. # indicates comparison with RF.

In the collagenase assay, all membranes underwent digestion in the presence of 1 CDU/mg (Collagen Digesting Units per milligram) collagenase at 37°C within a duration of 6 days (Figure 6). Evidently, the collagenase resistance of all crosslinked membranes exhibited a significant improvement.

The RF membrane experienced a mass loss exceeding 80% within the first hour of digestion, nearly undergoing complete degradation after 4 h. Notably, the HMDI crosslinking, while enhancing the collagenase resistance of the membrane, yielded the least improvement among the crosslinked membranes. Both the H1 and H2 membranes demonstrated notably higher mass losses than the other crosslinked membranes starting from the fourth hour of digestion (**p < 0.05, ***p < 0.001, ****p < 0.0001). After 6 days of collagenase digestion, these two groups retained less than 20% of their initial mass. It is worth mentioning that collagenase stability exhibited a slight increase with the elevation of crosslinker concentration from H1 (50 wt% HMDI) to H2 (100 wt% HMDI) (**p < 0.05, after 24 h of digestion).

Conversely, the remaining three crosslinked membrane types, i.e., the GA, EC, and PC membranes, showcased higher collagenase stability throughout the study, retaining over 80% of their initial mass up to 6 days of digestion, with no significant differences observed among them.

### 3.5 Histopathological results

#### 3.5.1 Comprehensive histological outcomes

**FIGURE 7:**
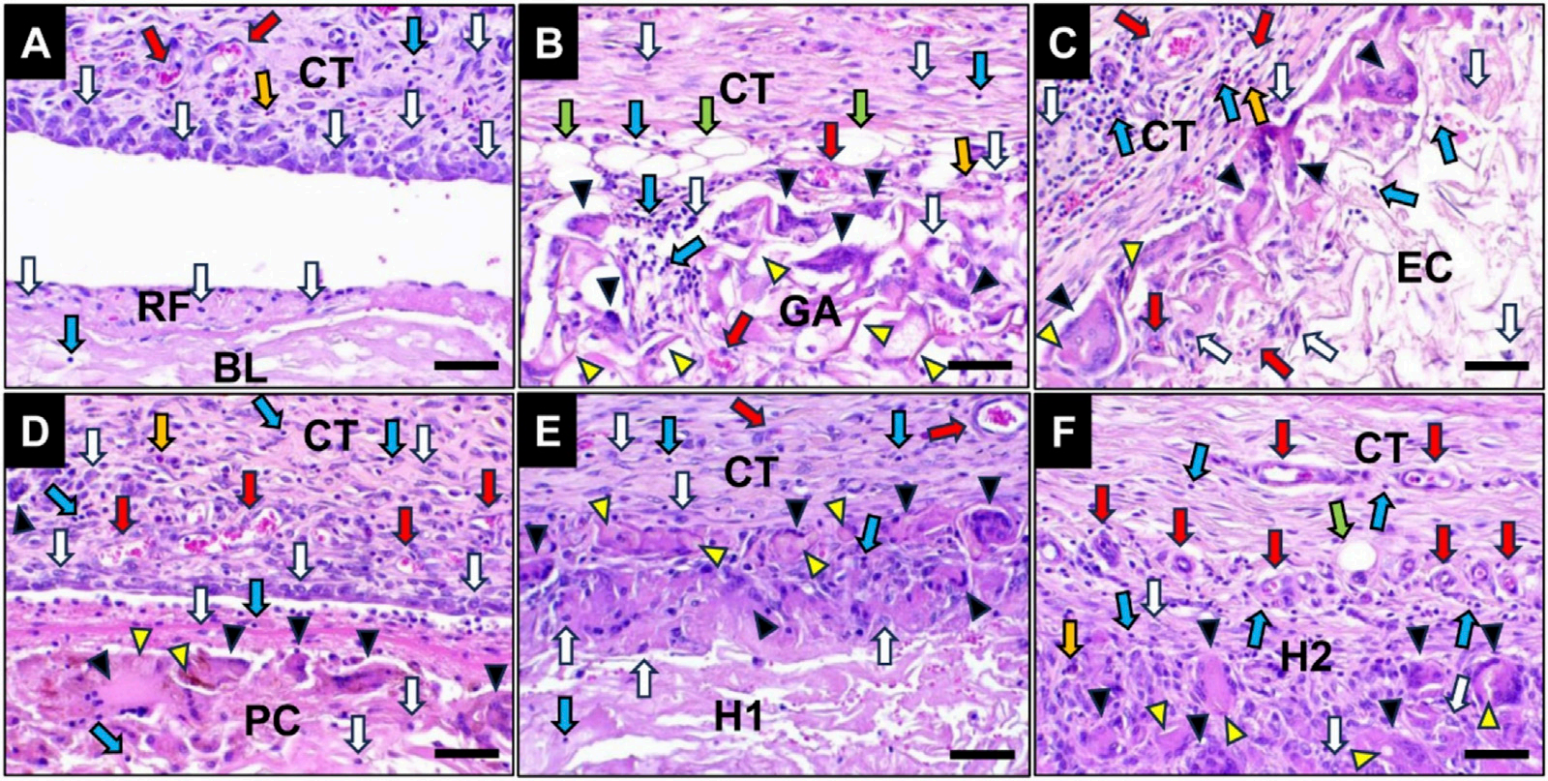
Exemplary histological images of the collagen membranes at 10 days post-implantation within the subcutaneous connective tissue (CT). **(A)** RF group, **(B)** GA group, **(C)** EC group, **(D)** PC group, **(E)** H1 group and **(F)** H2 group. White arrows: macrophages, blue arrows: lymphocytes, green arrows: fat tissue, yellow arrow: granulocytes, red arrows: vessels, yellow arrowheads: residual materials, black arrowheads: MNGCs, BL: base layer. (HE-stainings, 400x magnifications, scale bars: 20 µm).

The histopathological evaluation revealed that all cross-linked membranes remained detectable within the implant bed at 10 days post-implantation, eliciting an inflammatory tissue reaction (Figure 7). At this early study time point the RF membrane was already nearly completely degraded and thus observable in form of a thin layer on top of the base layer (Figure 7A). As expected, the RF membrane elicited a mild inflammatory tissue response involving mainly macrophages beside lower numbers of eosinophils and lymphocytes accompanied by a marginal neovascularization (Figure 7A).

In contrast, all of the crosslinked membranes induced a more pronounced inflammatory tissue response including moderate numbers of multinucleated giant cells (MNGCs) beside the afore-mentioned mononuclear cell types (Figure 7).

At 30 days post-implantation, the fleece layer of RF group was fully degraded and was thus unobservable (Figure 7A). In comparison to previous study time point, a very mild inflammation with a reduced number of inflammatory cells surrounding the base layer was detected in the RF group (Figure 7A).

**FIGURE 8:**
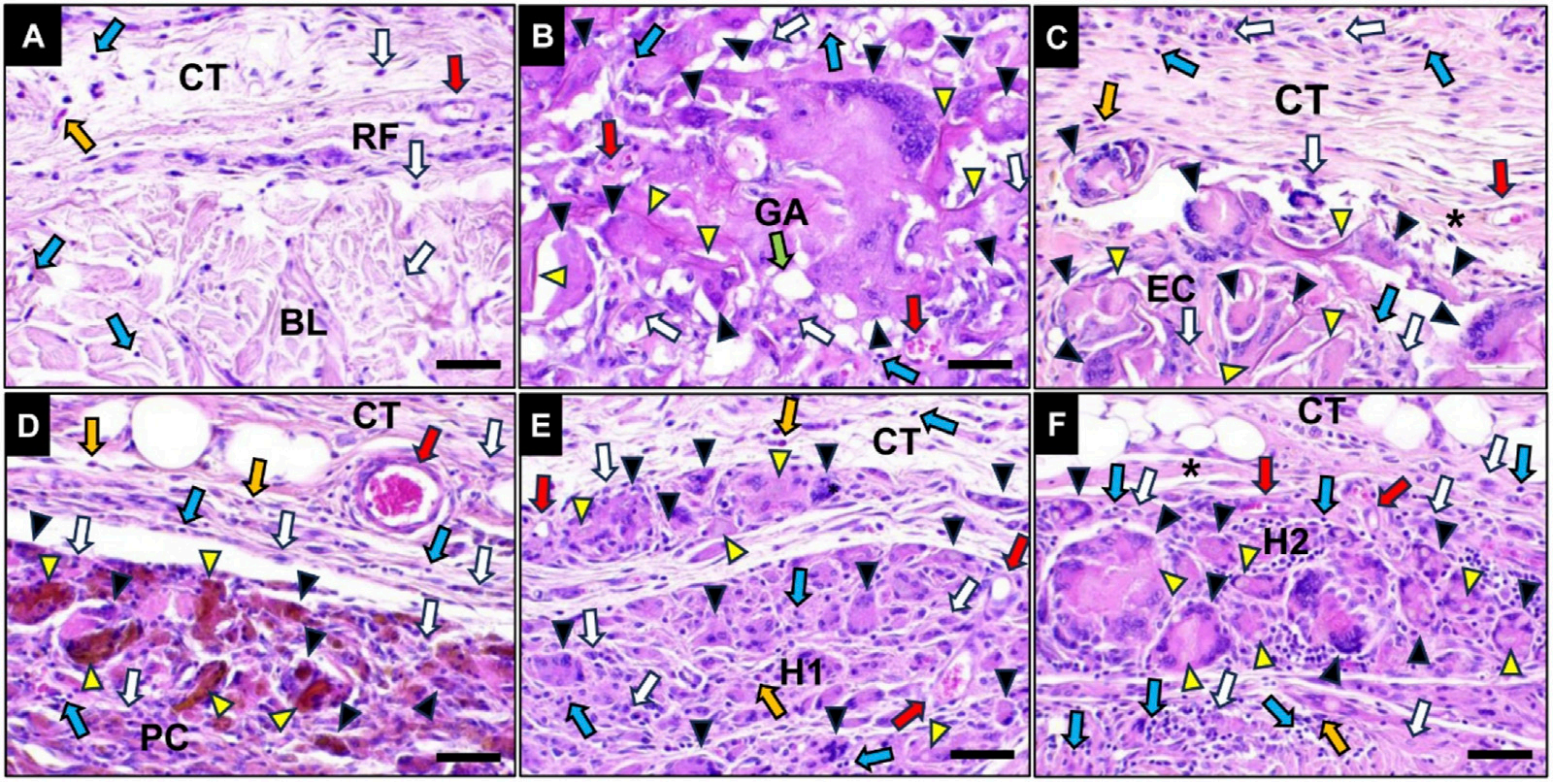
Exemplary histological images of the crosslinked collagen membranes at 30 days post-implantation within the subcutaneous connective tissue (CT). **(A)** RF group, **(B)** GA group **(C)** EC group, **(D)** PC group, **(E)** H1 group and **(F)** H2 group. White arrows: macrophages, blue arrows: lymphocytes, green arrows: fat tissue, yellow arrow: granulocytes, red arrows: vessels, yellow arrowheads: residual materials, black arrowheads: MNGCs, BL: base layer. (HE-stainings, 400x magnifications, scale bars: 20 µm).

Furthermore, a comparable inflammatory tissue response mainly involving MNGCs, macrophages and lymphocytes to that observed at day 10 post-implantation was detected in all cross-linked groups at day 30 post-implantation, with increased infiltration of reactive tissue into the membrane area (Figure 8). The presence of numerous membrane fragments surrounded by MNGCs and macrophages at the material-tissue interfaces especially in the GA, EC and H2 groups indicated that a fast phagocyte-driven material degradation was ongoing at this time point.

**FIGURE 9:**
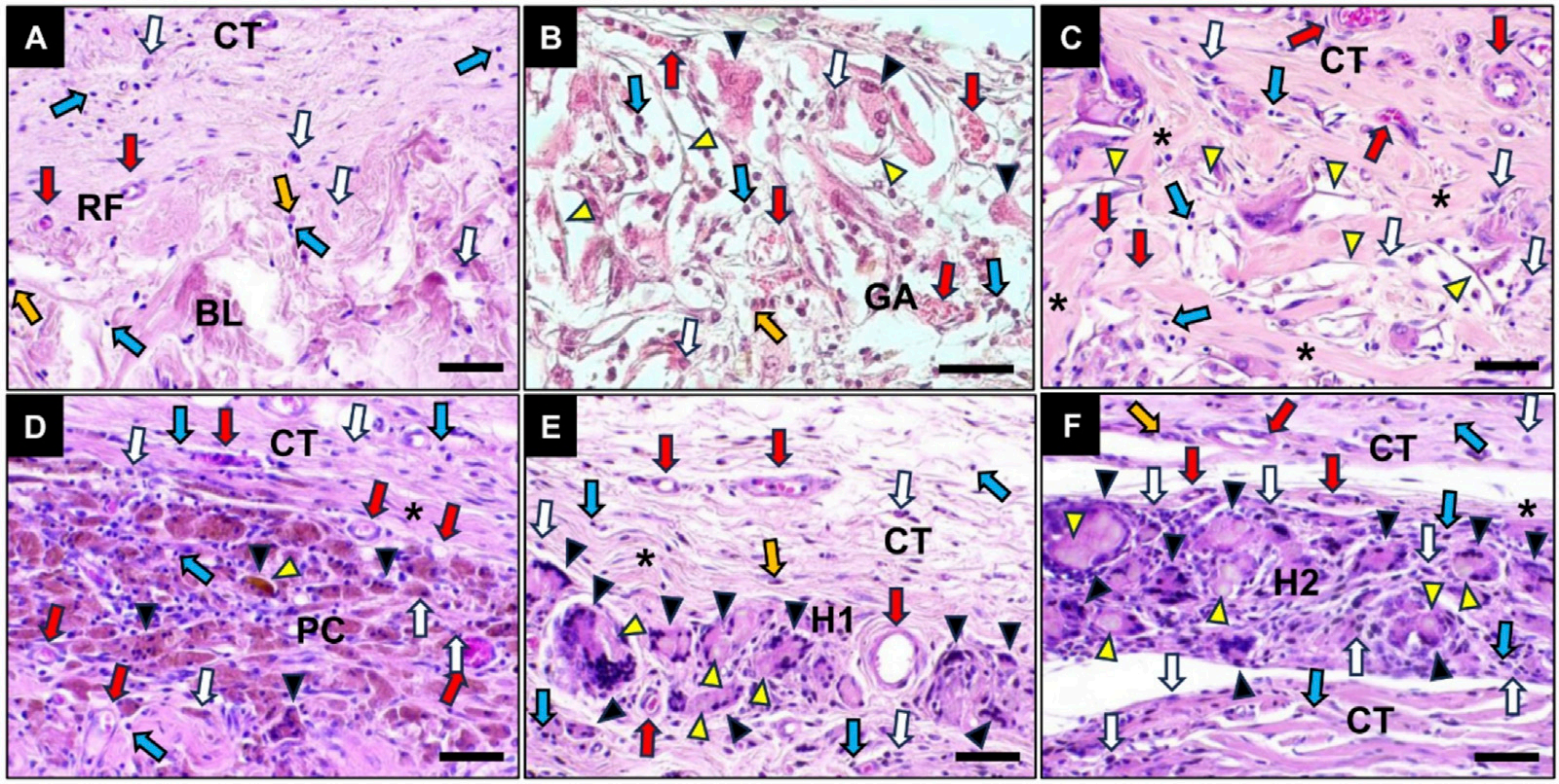
Exemplary histological images of the crosslinked collagen membranes at 90 days post-implantation within the subcutaneous connective tissue (CT). **(A)** RF group, **(B)** GA group **(C)** EC group, **(D)** PC group, **(E)** H1 group and **(F)** H2 group. White arrows: macrophages, blue arrows: lymphocytes, green arrows: fat tissue, yellow arrow: granulocytes, red arrows: vessels, yellow arrowheads: residual materials, black arrowheads: MNGCs, BL: base layer. (HE-stainings, 400x magnifications, scale bars: 20 µm).

At 90 days post-implantation, no histological signs of material-induced inflammatory tissue response in the RF group were detected as no signs of this layer were detectable (Figure 9A). However, material-induced inflammatory responses including mainly MNGCs, macrophages and lymphocytes were still clearly observed in all crosslinked groups and these membranes were nearly completely fragmented (Figures 9B–F). The histopathological evaluation showed that especially in the groups of H1 and H2 membranes, the membrane region was completely penetrated by MNGCs (Figures 9E, F). The connective tissue that was observable within the interspaces of the material fragments contained high numbers of small and medium-sized vessels. Furthermore, a slight fibrosis was observed surrounding the implanted materials in the PC, H1 and H2 membrane groups, while a higher extent of fibrosis surrounding all material fibers was found in the EC membrane group.

#### 3.5.2 Immune response

**FIGURE 10:**
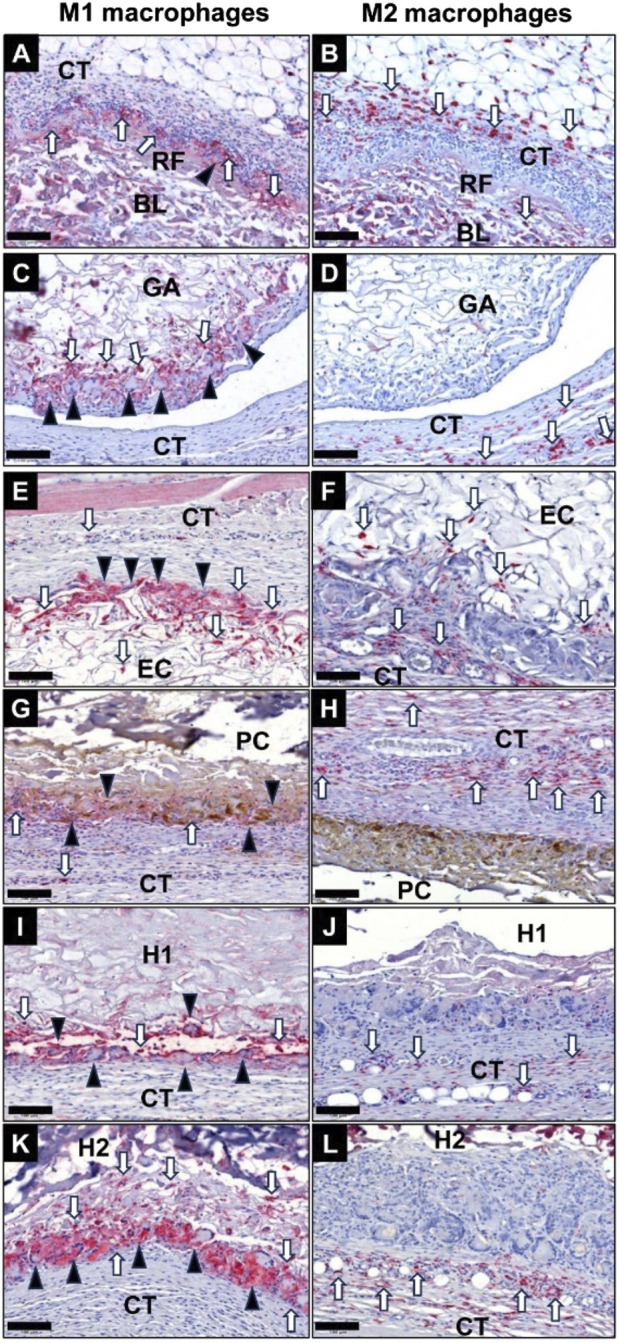
Exemplary images of the occurrence of pro-inflammatory M1 macrophages (left column) and anti-inflammatory M2 macrophages (right column) within the implantation beds of the different collagen membranes at 10 days post-implantation within the subcutaneous connective tissue (CT). **(A)** and **(B)** RF group, **(C)** and **(D)** GA group, **(E)** and **(F)** EC group, **(G)** and **(H)** PC group, **(I)** and **(J)** H1 group, **(K)** and **(L)** H2 group. Black arrowheads: MNGCs, white arrows: macrophages (CD11c- and CD163-immunostainings, 400x magnifications, scale bars = 100 μm).

The histological analysis of the occurrence of macrophages subtypes at 10 days post-implantation revealed that especially the membranes in the GA group, the EC group and the H1 group induced a more pronounced pro-inflammatory response (Figures 10C, E, I). In contrast a balanced ratio between M1 and M2 macrophages was already detected in the RF group, the PC group and the H2 group at this early stage (Figure 10). Furthermore, comparable low numbers of M2-macrophages were found in all study groups at this study time point. In all groups the MNGCs only showed signs of a CD11c-expression (Figure 10). Additionally, a spatial distribution pattern was observable in all study groups at this early study point as the pro-inflammatory phagocytes (M1-macrophages and MNGCs) were located in direct vicinity of the membranes, i.e., at the material-tissue surfaces or within the superficial membrane areas, while M2-macrophages were predominantly located in the periphery of the implantation beds of the membranes (Figure 10).

**FIGURE 11:**
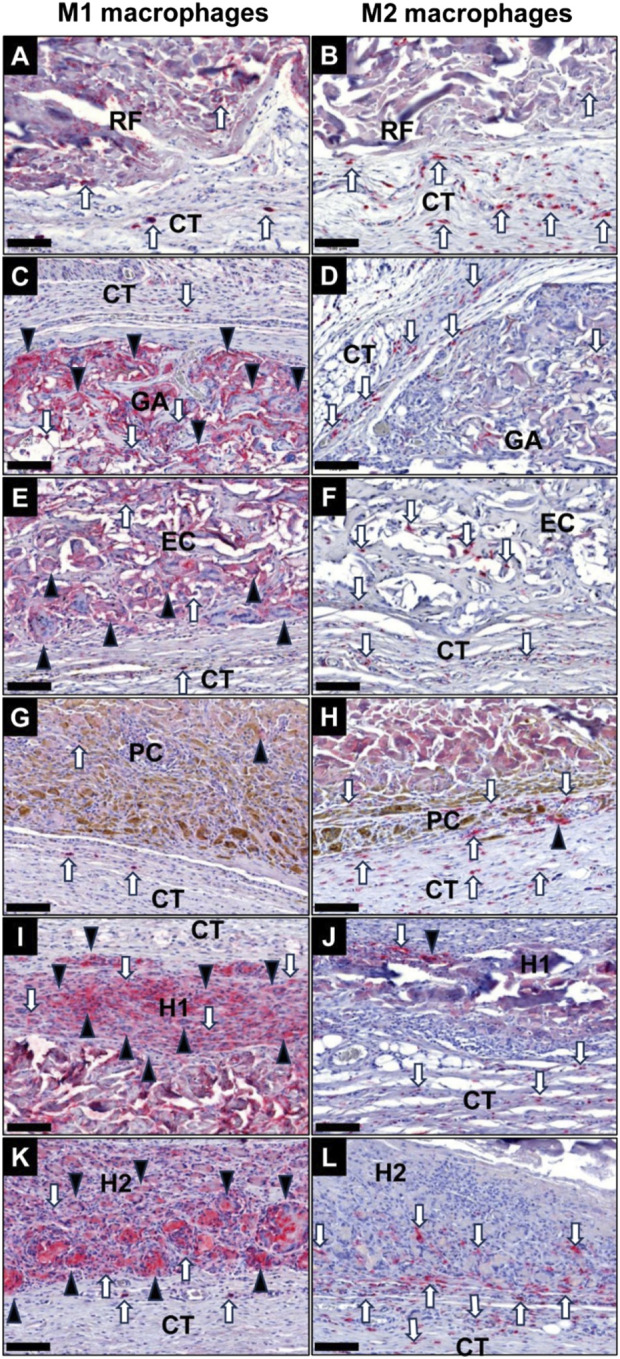
Exemplary images of the occurrence of pro-inflammatory M1 macrophages (left column) and anti-inflammatory M2 macrophages (right column) within the implantation beds of the different collagen membranes at 30 days post-implantation within the subcutaneous connective tissue (CT). **(A)** and **(B)** RF group, **(C)** and **(D)** GA group, **(E)** and **(F)** EC group, **(G)** and **(H)** PC group, **(I)** and **(J)** H1 group, **(K)** and **(L)** H2 group. Black arrowheads: MNGCs, white arrows: macrophages (CD11c- and CD163-immunostainings, 400x magnifications, scale bars = 100 μm).

At 30 days post-implantation, the highest numbers of M1-macrophages were detectable at the material-tissue-interfaces in the groups of the GA, EC, H1 and H2 membranes, which very visibly higher compared to the respective M2-macrophage numbers (Figure 11). In the groups of the RF and PC membranes lower M1-macrophage numbers were found, which were comparable to the respective anti-inflammatory subtype numbers. Only in the group of the RF membranes the numbers of anti-inflammatory macrophages seemed to be higher compared to the occurrence of M1-macrophages. However, comparable numbers of M2-macrophage were noticeable in all study groups at this intermediate study time point. In all groups of the crosslinked membranes CD11c-positive MNGCs were found within the implantation beds, while no MNGCs were detected in the RF group at this time point (Figure 11). Also, the afore-mentioned spatial distribution pattern of pro- and anti-inflammatory immune cells was observable in all study groups at this study point.

**FIGURE 12:**
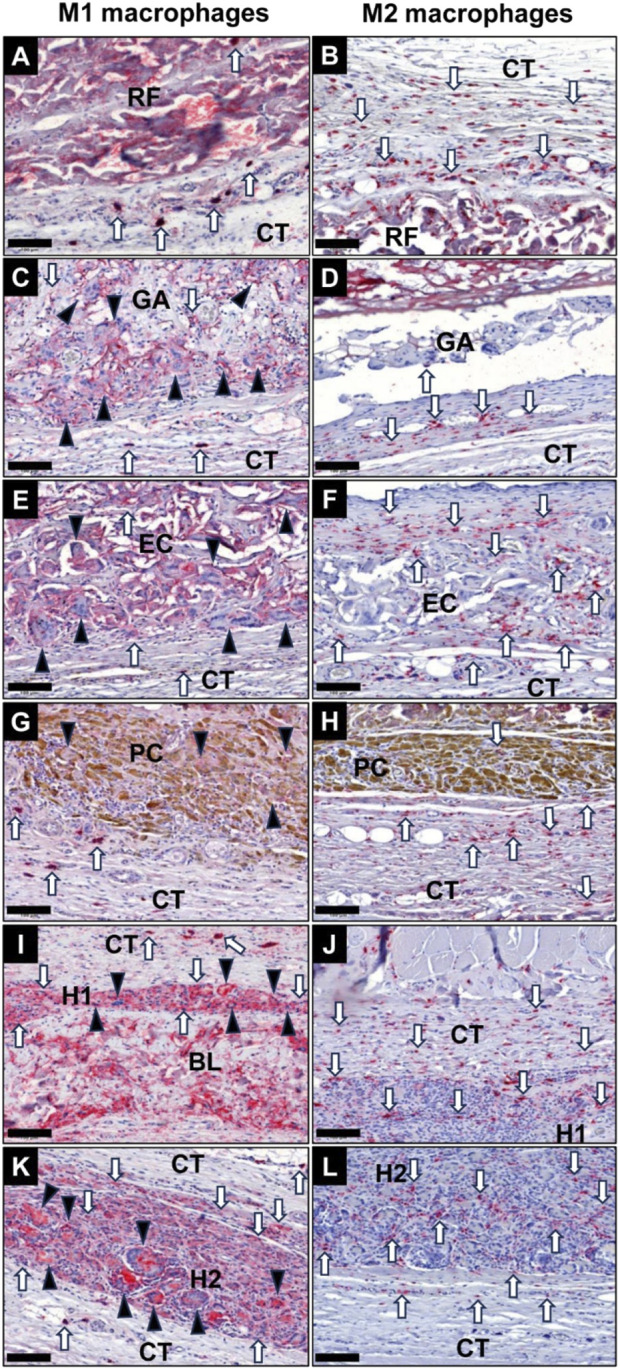
Exemplary images of the occurrence of pro-inflammatory M1 macrophages (left column) and anti-inflammatory M2 macrophages (right column) within the implantation beds of the different collagen membranes at 90 days post-implantation within the subcutaneous connective tissue (CT). **(A)** and **(B)** RF group, **(C)** and **(D)** GA group, **(E)** and **(F)** EC group, **(G)** and **(H)** PC group, **(I)** and **(J)** H1 group, **(K)** and **(L)** H2 group. Black arrowheads: MNGCs, white arrows: macrophages (CD11c- and CD163-immunostainings, 400x magnifications, scale bars = 100 μm).

At 90 days post-implantation, both the RF and PC membrane groups exhibited considerably lower levels of M1-macrophages and MNGCs compared to other experimental groups (Figure 12). The number of M2-macrophages in the RF and PC membrane groups was higher compared to the M1-macrophage numbers and predominantly located within the reactive inflammatory tissue surrounding the membranes. However, comparable M2-macrophage numbers were found in all study groups. In contrast, visibly higher presence of pro-inflammatory macrophages and MNGCs was detected in the groups of the GA, EC, H1, and H2 membranes, which were also higher compared to 30 days post-implantation. Furthermore, an increased number of M2-macrophages was detected within the invading connective tissue in all groups of the crosslinked membranes, while the afore-mentioned spatial distribution pattern of pro- and anti-inflammatory immune cells was in general still observable in all study groups.

#### 3.5.3 Vascularization pattern

**FIGURE 13:**
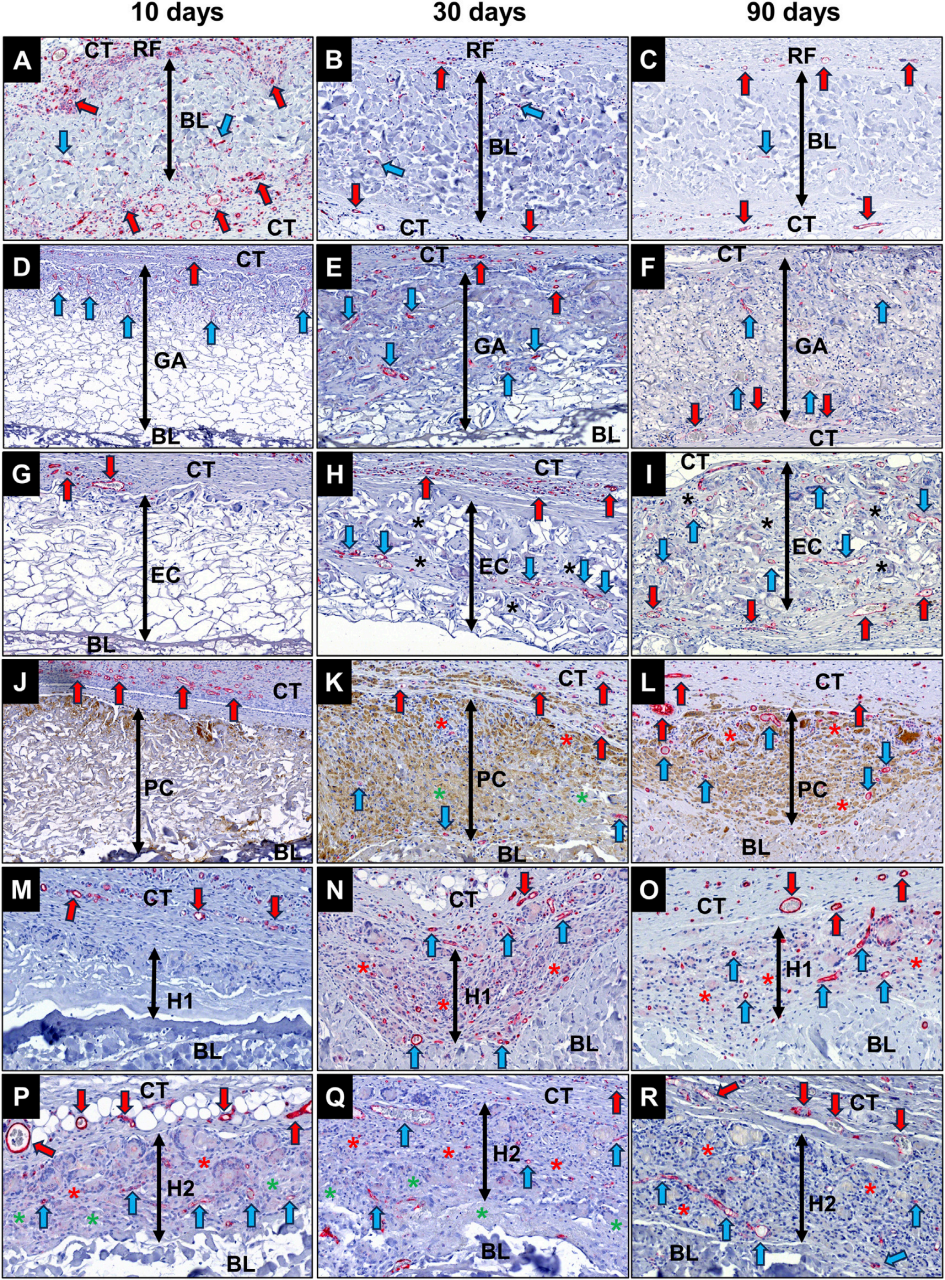
Exemplary images of the implantation bed vascularization of the different collagen membranes. **(A–C)** RF group; **(D–F)** GA group; **(G–I)** EC group; **(J–L)** PC group; **(M–O)** H1 group; **(P–R)** H2 group. Red arrows, vessels within the peripheral connective tissue, blue arrows, vessels within the membrane region, black asterisks, infiltrated connective tissue, green asterisks, residual materials, red asterisks, original location of the RF membrane. CT, connective tissue (CD31-immunostainings, 200x magnifications, excerpts of totals scans).

The histological analysis of the implantation bed vascularization revealed that the RF membrane area was nearly completely replaced by connective tissue (Figures 13A–C). Thereby, the membrane area as well the surrounding connective tissue showed a higher vascularization pattern, i.e., more small blood vessels with higher lumina, at 10 days post-implantation compared to the other study groups.

Stable porous membrane structures were observed in the groups of the GA, EC, and PC membranes at this early time point, accompanied by slight reactive tissue ingrowth. Due to limited tissue infiltration, blood vessels in these three groups were mainly observed in the surrounding connective tissue of the membrane. Although the H1 membrane was still detectable at this time point, the edge region of the membrane was completely degraded due to ingrowth of reactive tissue, with only a small amount of membrane fragments observable (Figure 13P). In the H2 group, inflammatory tissue penetration into the membrane was the most severe among all crosslinked membranes. The membrane area of H2 group was nearly completely infiltrated by macrophages and MNGCs, with abundant neovascularization observed in the infiltrated tissue (Figure 13P).

At the 30 days post-implantation, the membrane in the GA and EC groups exhibited extensive fragmentation, with connective tissue penetrating nearly completely through the membrane area (Figures 13E, H). Abundant undegraded collagen fibers and newly formed blood vessels were observable within the invading tissue. Conversely, in the PC, H1, and H2 groups, the membranes were completely fragmented at this time point, leaving behind only remnants of collagen fragments. The membrane area was entirely replaced by reactive tissue containing numerous newly formed blood vessels (Figures 13K, N, Q).

At 90 days following implantation, the membranes in the GA and EC groups exhibited complete fragmentation, while still retaining a notable presence of collagen fragments and fibers (Figures 13F, I). The inflammatory regions of PC, H1 and H2 groups had diminished compared to the 30-day post-implantation period, leaving behind minimal remnants of collagen fragments (Figures 13L, O, R). Consistent with former study time points, a substantial vascular network was evident within the invading connective tissue associated with the material in all research groups.

### 3.6 Histomorphometrical results

#### 3.6.1 Occurrence of macrophage subtypes

**FIGURE 14:**
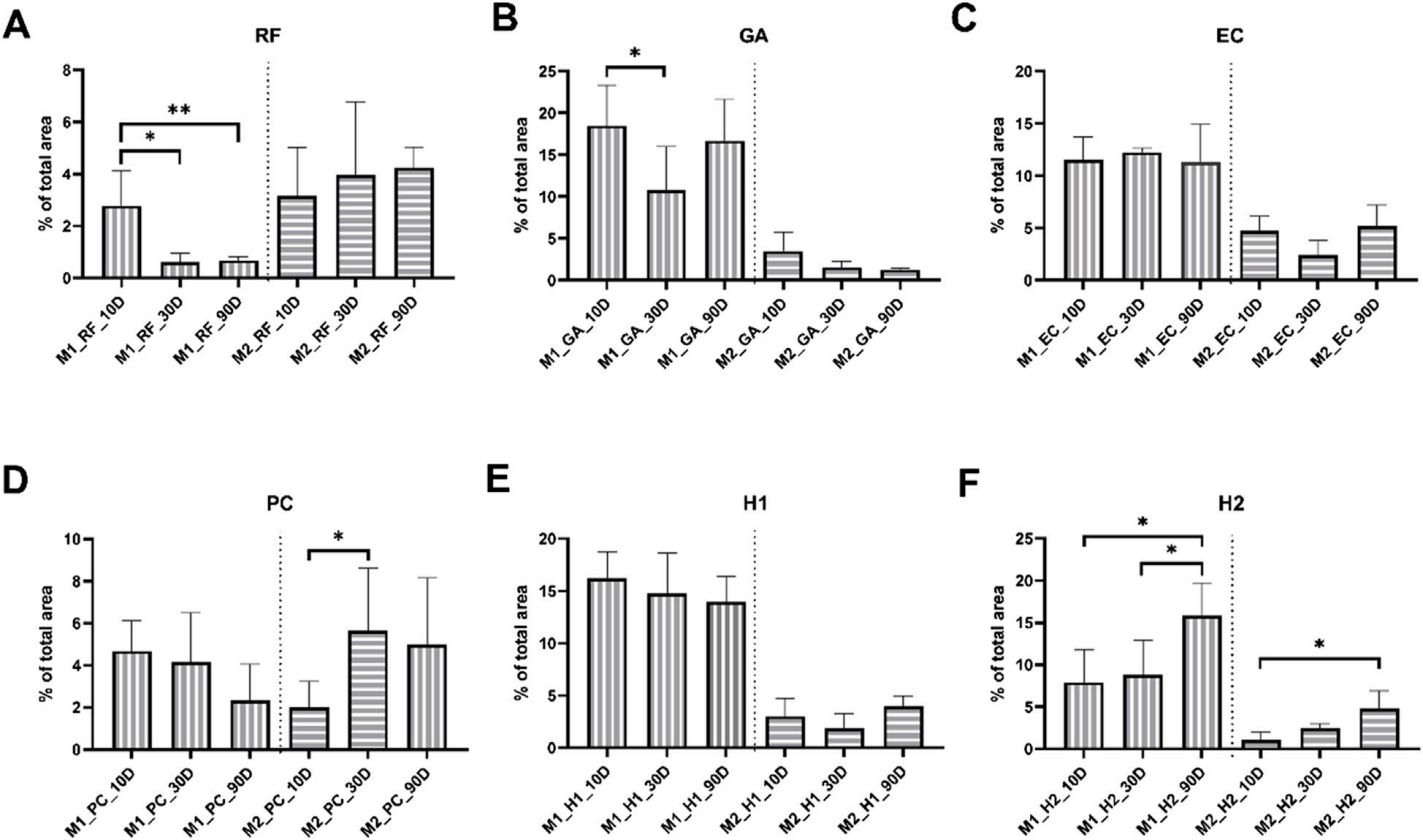
The intraindividual comparison of the histomorphometrical results of the macrophage response to the tested collagen membranes **(A-F)**. Intraindividual significances: *p < 0.05, **p < 0.01.

The histomorphometrical analysis of the macrophage subtype occurrence revealed that the RF membrane induced an immune response mainly dominated by M2-macrophages (Figure 14A). Within this group, the numbers of M1-macrophages reached a peak at 10 days post-implantation followed by a significant decrease at day 30 and day 90 post-implantation (*p < 0.05). In contrast, the numbers of the M2-macrophages remained on a constant niveau throughout the implantation period showing a minimal trend towards an increase during the time course of the study.

Throughout the entirety of the implantation period, the levels of M1 macrophages induced by the PC group were very similar compared to the values in the RF membrane group (Figure 14D). Thus, a decreasing tendency in the numbers of M1-macrophages was found starting with day 10 post-implantation up to the end of the observation period at day 90 post-implantation. In contrast, the numbers of M2-macrophages increased during this time frame with a significant increase between day 10 and 30 post-implantation (*p < 0.05).

However, with exception of the PC membrane, all crosslinked membranes induced an immune response strongly dominated by M1-macrophages. The M1-macrophage response in the GA group also exhibited notable changes, with cell numbers significantly lower at 30 days post-implantation compared to 10 days (*p < 0.05), followed by a subsequent increase at 90 days post-implantation (Figure 14B). In the groups of the EC and the H1 membranes no changes of the M1-or M2-macrophage numbers were found during the time course of the study (Figures 14C, E).

In contrast, the H2 membrane induced significantly lower M1 macrophage levels in the early post-implantation phase compared to the later phase (*p < 0.05) (Figure 14F), while also an increase of M2 macrophages over the study period with a significant difference between day 10 and 90 post-implantation (*p < 0.05) was found.

**FIGURE 15:**
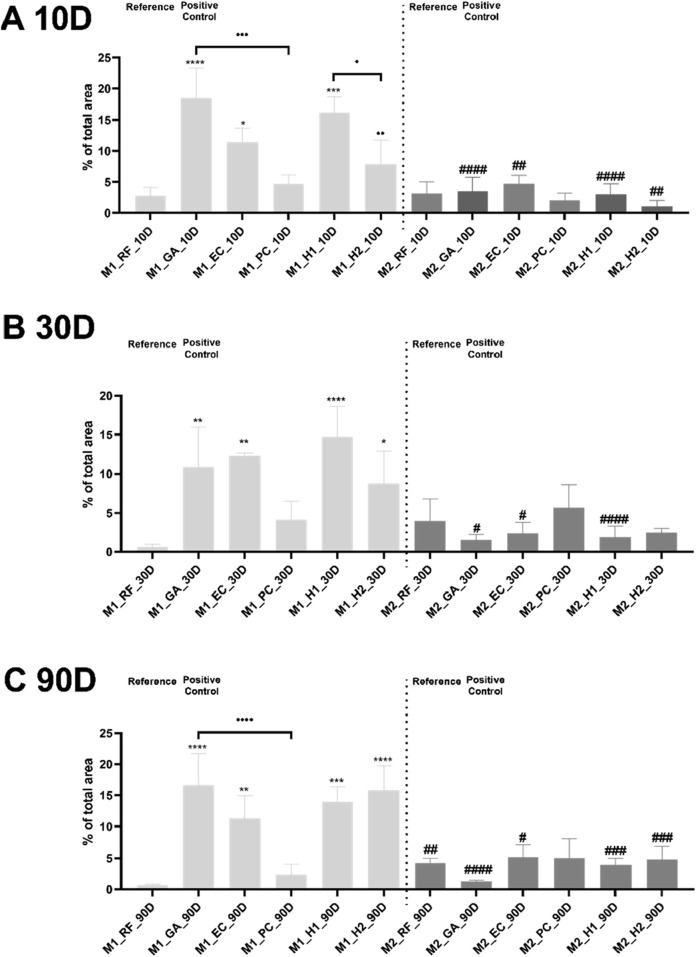
The interindividual comparison of the histomorphometrical results of the macrophage subtype response to the tested collagen membranes. Statistical interindividual significances: */^#^p < 0.05, /^##^ *p* < 0.01, /^###^ *p* < 0.001, **/. . . ./^####^ *p* < 0.0001. indicates comparison with RF, ^#^indicates intraindividual significance between M1 and M2.

At 10 days post-implantation, the GA membrane as positive control triggered a significantly higher M1-macrophage level compared with the RF membrane (****p < 0.0001) (Figure 15A). Apart from the values in the GA membrane group, the values of levels of M1 macrophages in the H1 and EC membrane groups were also significantly higher than in the RF group (***p < 0.001, *p < 0.05). In contrast, the PC and H2 membranes exhibited significantly lower M1 levels than the GA membrane (^…^ *p* < 0.001, ^..^ *p* < 0.01). Notably, at this time point, all crosslinked membrane induced M2 macrophages levels significantly lower than M1 levels (^#^ *p* < 0.05, ^##^ *p* < 0.01, ^###^ *p* < 0.001, ^####^ *p* < 0.0001) with exception of the values in the RF group that were comparable.

At 30 days post-implantation, except for the PC membrane, all crosslinked membrane exhibited significantly higher M1 levels than in the RF membrane group (*p < 0.05, **p < 0.01, ****p < 0.0001) (Figure 15B). Notably, the H1 membrane induced the highest M1-macrophage response, even slightly surpassing the values in the GA membrane group. Although without significance, the PC membrane induced the highest M2-macrophage response out of all crosslinked membranes at this timepoint. Furthermore, the GA and H1 membranes induced M2-macrophage values that were significantly lower than the M1-macrophage levels (^#^ *p* < 0.05, ^####^ *p* < 0.0001).

At 90 days post-implantation, the PC membrane continued to exhibit the lowest M1 levels among all membranes (Figure 15C). All crosslinked membranes induced significantly higher M1-macrophage responses than the RF membrane (**p < 0.01, ***p < 0.001, ****p < 0.0001). At this timepoint, the M2-macrophage level in the RF membrane group was significantly higher than the respective M1-macrophage level (^##^ *p* < 0.01). In the PC group, the low M1-and M2-macrophage levels did not show statistical significance. However, in the other crosslinked membrane groups, the M2-macrophage values remained significantly lower than the respective M1-macrophage levels (^#^ *p* < 0.05, ^###^ *p* < 0.001, ^####^ *p* < 0.0001).

#### 3.6.2 Occurrence of multinucleated giant cells (MNGCs)

**FIGURE 16:**
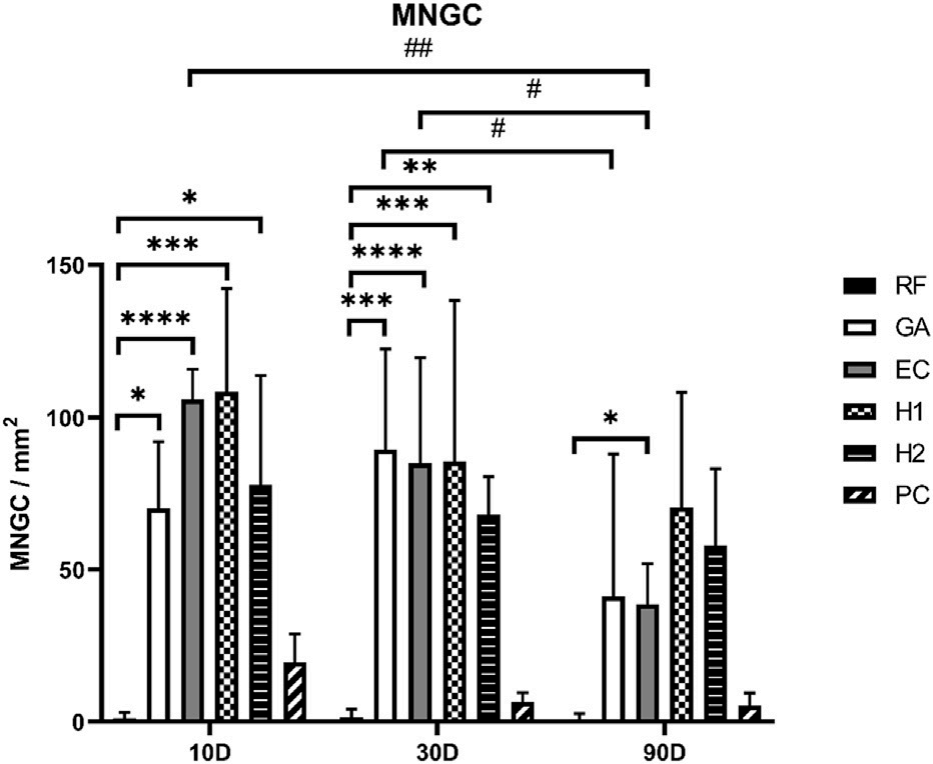
The histomorphometrical results of MNGCs within the implanted bed of the tested collagen membranes. The means are presented with error bars representing standard deviations. Statistical significance: */#*p* < 0.05, **/##*p* < 0.01, ***p < 0.001, ****p < 0.0001.

Multinucleated giant cells (MNGCs), considered a pivotal immunological and biodegradation indicator, were also analyzed in this study. As shown in Figure16, MNGCs triggered by the RF (reference) membrane consistently maintained very low levels throughout whole study period.

The PC membranes induced slightly more MNGCs compared to the numbers in the RF membrane group without significant differences. In contrast, all the other crosslinked membrane induced significant higher MNGC numbers compared to that found in the group of the RF membranes at day 10 and 30 post-implantation (*p < 0.05, **p < 0.01, ***p < 0.001, ****p < 0.0001). At 90 days post-implantation, significant differences were only observed between the values in the EC membrane group and the RF membrane group (*p < 0.05).

Furthermore, intra-individual significant differences in the MNGC numbers in the GA and EC groups were also observed at different intervals. The quantity of MNGCs in the EC group exhibited a declining trend throughout the implantation period, with MNGC numbers that significantly decreased between 10- and 90-day post-implantation (##*p* < 0.01) but also between 30- and 90-day post-implantation (#*p* < 0.05). Similarly, in the GA group, the level of MNGCs at 90 days post-implantation was also significantly lower than at 30 days post-implantation (#*p* < 0.05).

#### 3.6.3 Vascularization

The vascularization within implantation area including the intra-membrane (IMR) and peri-membrane (PMR) regions, was examined focusing on two key parameters: vessel number/mm^2^ (vessel density) and vessel area in % (vessel area fraction).

**FIGURE 17:**
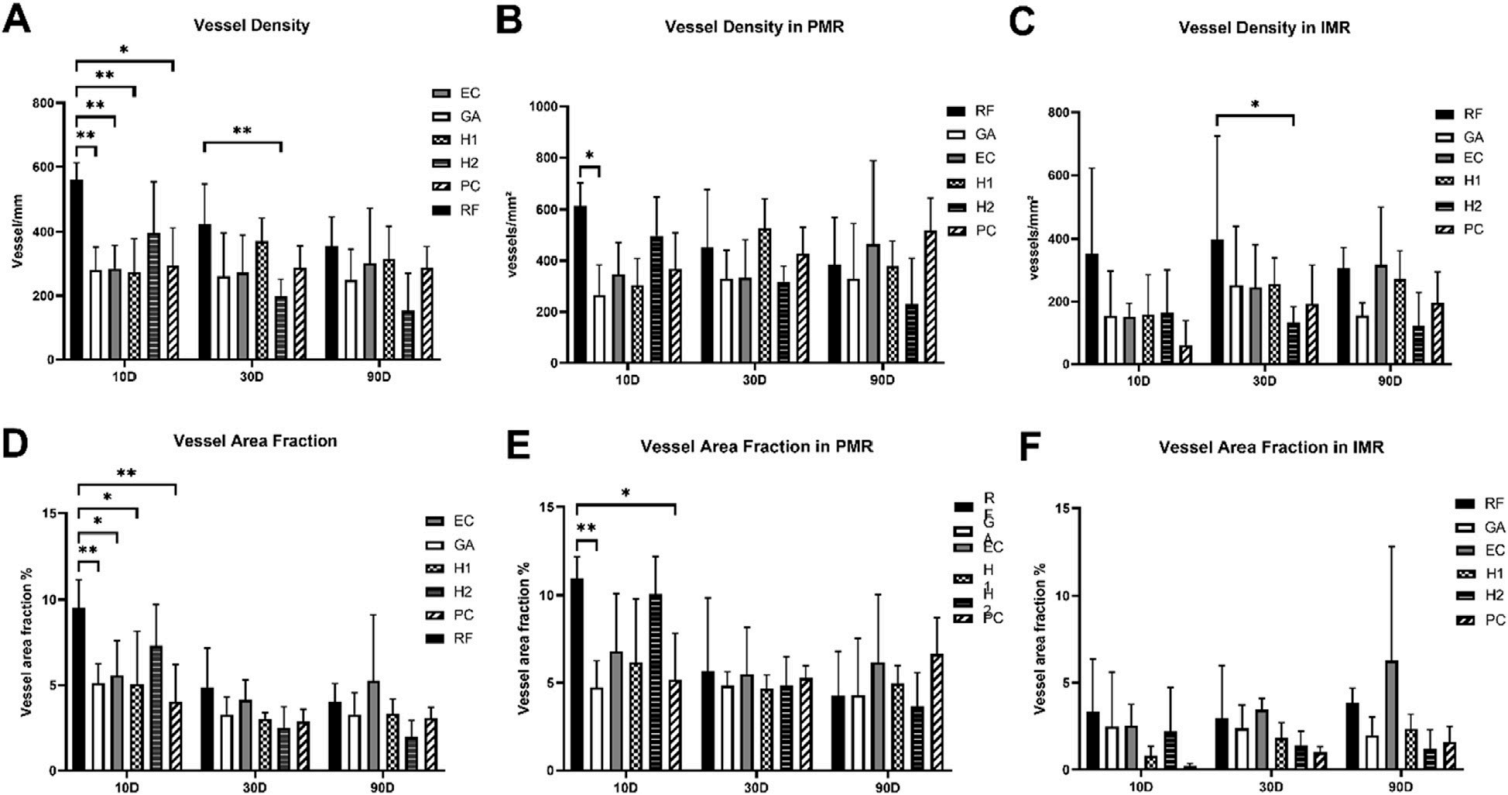
The histomorphometrical results of the *(A-C)* vessel density and *(D-F)* vessel area fraction measurements within the implanted beds of the tested collagen membranes. PMR: peri-membrane regions. IMR: intra-membrane region. Statistical interindividual significance: *p* < 0.05, *p < 0.01.

At 10 days post-implantation, the RF group exhibited the highest overall vessel density and vessel area fraction, as the values in this group were significantly higher than in the EC, GA, PC, and H1 groups (*p < 0.05, **p < 0.01) (Figures 17A, D). Thereby, only significant differences were found within the peri-membrane regions, while no significances were detectable within the intra-membrane areas. Thus, both the vessel density and vessel area fraction in the PMR area of GA group were significantly lower than that of the RF group (*p < 0.05, **p < 0.01) (Figures 17B, E). Also, the vessel area fraction in PMR area of the PC membrane group was significantly lower than that in the RF group (*p < 0.05) (Figure 17E). Furthermore, no significances between all groups were found within the intra-membrane region at this early study time point (Figures 17A–F).

At 30 days post-implantation, only the overall vessel density in the H_2_ membrane group was significantly lower than that observed in the RF group (**p < 0.01) (Figure 17A). Apart from the H2 group, no intergroup significances were observed at 30 days post-implantation. Also, no differences in the overall vessel area fraction were observed (Figure 17B). Additionally, the analysis showed that no differences nor in the vessel density or the vessel area fraction between all study groups within the peri-membrane area were found at this study point (Figures 17A–F). Only, significantly lower vessel density values were found in the H2 membrane group compared to the values in the RF membrane group within the intra-membrane region were found (*p < 0.05) (Figure 17C), while no other differences were calculated.

At 90 days post-implantation, no significant differences in the overall vascularization parameters nor in the different regions of the implantation beds were found between all study groups (Figures 17A–F).

## 4 Discussion

Barrier membranes were initially designed to form a physical barrier between soft or gingival tissue and the jaw bone defect to prevent the migration of cell types (such as epithelial cells) that might negatively interfere with the bony healing process during Guided Bone Regeneration (GBR) procedures (Yang et al., 2022). By creating a secluded space, the optimal membrane should allow specific cells such as osteoblasts and precursor cells to populate the defect site and promote the regeneration of the bone tissue (Allan et al., 2021; Ren et al., 2022). During excessive development of resorbable barrier membranes in the last decades this material class is nowadays mainly composed of xenogeneic collagen of different animal sources and tissue origins, while the optimum standing time has been determined: Resorbable barrier membranes require integrity for 4–6 weeks for periodontal tissue regeneration and 16–24 weeks for bone tissue regeneration (Caballé-Serrano et al., 2019). However, native collagen derived from porcine skin that is mainly used as tissue source falls short of meeting the requirement. In this context, chemical crosslinking of native collagen is a well-established approach to improve the mechanical properties as well as the standing time of such materials and has a long history with respect to medical applications (Oryan et al., 2018; Adamiak and Sionkowska, 2020).

Interestingly, the requirements for this type of biomaterial have also changed considerably in recent years. Thus, the requirement for a purely physical barrier functionality has been expanded to include various biological functionalities. This topic was discussed even in the last years and especially “bioactive properties” have been discussed in this context (Omar et al., 2019; Ren et al., 2022; Yang et al., 2022). Altogether, this term summarizes that the optimal barrier membrane should “actively” support the process of bone-healing, which includes also the establishment of an adequate angiogenesis and vascularization for bone tissue regeneration in form of a so-called “transmembraneous vascularization” but furthermore an optimal inflammatory tissue response to support the underlying bone healing process. In this context, Alkildani and colleagues showed in an *in vivo* study that a native collagen-based barrier membrane was found to get ossified, while inducing a high occurrence of both M1-and M2-macrophages within its implantation bed significantly dominated by the anti-inflammatory subtype. More interesting, a correlation between M2-macrophages induced by the membrane and bone regeneration in the underlying bone defects was found that hints on the interaction of the immune responses induced by the collagen membrane and the bone healing process (Alkildani et al., 2023). These results and different other study results led to the realization that it is of great importance to understand the immunomodulatory features of collagen-based barrier membranes for their future development.

In the present study, the material and biological properties of four collagen membranes modified by different crosslinkers, i.e., GA, EC/NHS, PC, and HMDI, were analyzed via established *ex vivo*, *in vitro* and *in vivo* methodologies (Kapogianni et al., 2021; Radenković et al., 2021; Alkildani et al., 2023). These chemical crosslinking were attained via different interhelical linkages such as imine/amine bonds, amide bonds and hydrogen bonds (Tanzer, 1973; Adamiak and Sionkowska, 2020). In this context, it has already been described that GA is the most representative aldehyde crosslinker which reacts with lysine or hydroxylysine residues forming an intermediate Schiff base and its further reaction pattern leads to the formation of more stable covalent imine bonds (Ruijgrok et al., 1994; Zhang et al., 2022). However, the application of GA has limited in commercial bio-products due to the evidence that unreacted GA residues and by-products such as Schiff bases can induce cytotoxicity, inflammation, and calcification (Zhang et al., 2022). The *in vitro* study also supports above conclusion that the GA membrane exhibited high deviations from the non-cytotoxicity ranges and can thus be classified as cytoincompatible. Particularly in the BrdU assay, the values of the GA membrane were extremely low and not significantly different from the positive control (RM-A). Given this concern, EC/NHS gain a growing interest as the agent itself is not incorporated into the network after crosslinking (Yang, 2012). EC activates carboxylic acid groups on collagen to form an O-acylisourea intermediate, which then react with amino groups to create covalent amide bonds (Gao et al., 2022). After reaction, EC is transformed into water soluble urea derivatives which is easy to be removed. Another agent of interest is HMDI, which reacts with amino groups (-NH_2_) on their lysine residues of collagen through isocyanate groups to form stable urea linkages which is highly resistant to hydrolysis (Olde Damink et al., 1995; Sarrigiannidis et al., 2021). Unreacted HMDI and its byproducts, such as urethanes, are easier to remove and exhibit lower cytotoxicity compared to that of GA. Proanthocyanidins (PC), characterized by their anti-inflammatory properties and abundance of hydroxyl groups, serve as another beneficial cross-linking agent proficient in establishing hydrogen bonds with collagen molecules (Green et al., 2010; He et al., 2011; Vidal et al., 2016). In this study, collagen membranes prepared with the above three crosslinkers demonstrated satisfactory cytocompatibility and then following the 3R-cascade, implanted into subcutaneous connective tissue to determine the integration behavior, the standing time and the related immune responses as previously described (Ghanaati et al., 2010; Lindner et al., 2020; Naenni et al., 2020). GA membranes and RF membranes were employed as the positive control and negative control, respectively, in the further *in vivo* study.

Initially, the histological and the material analyses of the pure collagen membranes showed that all membranes displayed a well-defined layer characterized by a honeycomb-like pore structure. While only slight structural alterations occur following cross-linking, the introduction of all crosslinkers enhanced the tensile strength of collagen membranes by creating additional chemical bonds between collagen molecules. Notably, proanthocyanidins and hexamethylene diisocyanato (HMDI) crosslinked membranes exhibited similar ductility and denaturation temperature with RF membrane. However, both GA and EC membranes displayed decreased ductility, particularly the elongation at break of the EC membrane was notably lower than that of the PC membrane, which means that they may experience a propensity for lesser plastic deformation compared to the other membranes. Histological images showed consistent results that GA and EC membranes retained a bilayer structure with well-defined and interconnected pores and void spaces within the collagen matrix even after 30 days post-implantation. This stiff structure provides ample strength endure the compression and tension exerted by surrounding soft tissues and influencing the ability of membranes to absorb and retain water. This inference is further corroborated by the significantly increased swelling ratio observed in both EC and GA membranes compared to the rest groups. These two groups also displayed a stronger resistance to temperature which may indicate the forming of more robust molecular bridges between collagen fibers. These bridges contribute to a more interconnected and reinforced structure, making it harder for the collagen fibers to unravel during denaturation at high temperatures.

Moreover, the formation of molecular bridges serves to conceal collagen’s cleavage sites, thereby augmenting collagen’s resistance to enzymatic degradation (Oryan et al., 2018; Sarrigiannidis et al., 2021). The findings indicate that all crosslinked collagen membranes display significantly improved collagenase resistance, especially GA, EC and PC groups, which retained membrane integrity after 7 days of collagenase digestion *in vitro*. However, both H1 and H2 membranes crosslinked with Hexamethylene diisocyanate (HMDI) exhibited a significant mass loss after 4 h of collagenase digestion, persisting throughout the entire assay. Besides, the collagenase resistance of collagen materials is strongly influenced by the crosslinking protocol, such as concentration, crosslinking time and temperature (Adamiak and Sionkowska, 2020). Therefore, it is anticipated that optimizing the crosslinking scheme for HMDI-crosslinked membranes will enhance their enzyme resistance, which is supported by the fact that H2 crosslinked membrane, treated at a higher HMDI concentration, exhibited significantly higher mass residues compared to H1 crosslinked membrane after 24 h of collagenase digestion. Even digestion results of the GA membranes confirm the good suitability of this crosslinker - even apart from the problem of biocompatibility issues that can occur.

The results of the swelling ratio test showed that the EC, PC and GA membranes exhibited a significantly faster swelling rate and/or higher swelling ratio in comparison to the RF membrane. The H1 membrane exhibited a swelling pattern most similar to the RF membrane, whereas the H2 membrane, crosslinked by a higher HMDI concentration, displayed the lowest swelling rate and swelling ratio. This result is very important as it has been reported that crosslinked collagen membranes have prolonged degradation times, but their application is associated with significantly higher membrane exposure rates of up to 70.5% (Friedmann et al., 2011; Bouguezzi et al., 2020) Thereby, the premature exposure of membranes is often associated with bacterial invasion, as demonstrated by Becker et al. (2009) Even in view of the results won via the collagenase assay it can be concluded that the GA, EC and PC membranes might not be suitable for GBR procedures in patients with a thin gingiva biotype (Sarma et al., 2021), while the HDMI-crosslinked membranes might be more suitable even in this point.

The analyses revealed that the non-crosslinked reference membrane was degraded very fast until day 30 post implantation inducing a very low immune response mainly based on macrophages and without occurrence of multinucleated giant cells (MNGCs). Initially, M1-like macrophages were activated and displayed a pro-inflammatory phenotype during early implantation stages–mainly induced by the surgery and the related tissue defect. However, by day 30 post-implantation, the population of M1 macrophages notably diminished, giving way to a predominance of anti-inflammatory M2 macrophages. This result was to be expected and also corresponds to the results of various other studies (Ghanaati, 2012; Al-Maawi et al., 2018). This expected degradation rate, and the integration behavior were therefore also the reason for selecting this membrane as a negative control and show especially the fast biodegradation, which is obviously too fast for many GBR procedures. However, this results even in view of the immunological tissue response is very interesting as it has manifoldly discussed and shown that synthetic but also different other biomaterials such as bone substitutes induce a special spatiotemporal macrophage response (Abels et al., 2021; Barbeck et al., 2022; Barbeck et al., 2023; Ren et al., 2024). In this context it has been described that M1 macrophages even in case of “unnatural” materials are located at the material surfaces in combination with MNGCs as correlate of a foreign body response that is involved in material degradation via phagocytosis (Barbeck et al., 2023). In this context, the observed tissue response of the RF membrane is in complete contrast with this pro-inflammatory tissue response and might show that this implant material or the collagen fibers has a natural constitution and is more digested by mononuclear cells of the collagen turnover as described in case of the BioGide membrane (Ghanaati, 2012; Radenković et al., 2021). As the most widely used membrane in clinical practice, BioGide has been shown to involve only controlled tissue infiltration by mononuclear macrophages, which represents a beneficial tissue integration pattern for guided bone regeneration (GBR) membranes. This means that RF membrane rightly serves or can serve as a reference material for collagen-induced cellular transition in this study.

In contrast, the GA-crosslinked membrane, which was included into the study as a negative control group, induced a pronounced inflammatory tissue response including high numbers of MNGCs but was in total present until day 90 post-implantation. Thereby, mostly single fiber bundles surrounded by mononuclear and multinucleated phagocytes, which had spread over the entire implantation area, were found starting with day 30 post-implantation. This immune response is definitely more pro-inflammatory in nature by inclusion of higher numbers M1-macrophages and M1-MNGCs reflecting the “unnatural physicochemical material properties”, revealing that his membrane served also as a “negative reference material” Thus, this integration pattern is not in line with a real barrier functionality without cell infiltration or with minor cell infiltration as in the case of PTFE-based materials or other collagen-based membranes (Korzinskas et al., 2018; Ottenbacher et al., 2021; Radenković et al., 2021). In this context, it was shown that also a cell infiltration into the membrane structures of the currently most clinically used barrier membranes, i.e., Jason membrane and BioGide, was observable (Ottenbacher et al., 2021). However, the cell invasion in these latter cases only included macrophages and did thus not evoke such a high degradation pattern combined with a material fragmentation. Moreover, a recently published study by Alkildani *et al.* showed that the pericardium-based Jason membrane has a long-standing time and was completely remodeled into bone tissue within several weeks after implantation by serving as an osteoconductive scaffold (Alkildani et al., 2023). This leads to the conclusion that in the case of collagen-based membranes, cellular infiltration does not necessarily have to be harmful in terms of GBR functionality. As in the case of any (biodegradable) biomaterial, the material-specific tissue response, i.e., the cellular elements induced, in combination with the material properties, determines the degradation mechanisms and duration of biodegradation. The degradation behavior of the GA membrane seems thus represent not a suitable integration pattern.

In view of the integration and degradation pattern of the other crosslinked collagen membranes it was shown that the EC membrane also showed an invasion of M1-macrophages and high M1-MNGC numbers combined with a degradation pattern comparable to the GA membrane group. Histopathological analysis demonstrated that at 30 days post-implantation, the porous structure of EC membranes was still clearly visible. Subsequently, the material underwent collapse until the conclusion of the study. Interestingly, the collagen fibers were surrounded by a fiber-rich connective tissue with lower cell numbers and high vascularization, which might be a factor that can prevent the ingrowth of other tissues such as epithelial tissue and might thus be an interesting material candidate in the GBR procedure with a completely different approach. However, fibrosis is the end stage of the foreign body response to a biomaterial (Anderson et al., 2008) – meaning that the recipient tissue and the immune system of the recipient organism see no other option than to completely encapsulate the biomaterial and no longer allow any further interactions. It can therefore also be assumed in the case of this material candidate that it will not be possible to successfully develop a GBR membrane on this basis.

In the case of the membranes cross-linked with both the lower and higher HDMI concentration, this disintegration and degradation pattern was already observed on day 30 post-implantation - combined with high M1-macrophage and M1-MNGC numbers. On day 90, only cell-rich connective or granulation tissue was left and only very scarred membrane fragments, so that no barrier functionality was present in these two groups either. This outcome can be attributed to the lower resistance of H1 and H2 membranes to cell-secreted degrading enzymes, consistent with the findings from the previously described *in vitro* collagenase experiments. It is noteworthy that although the collagenase resistance of H2 membranes appeared to be superior to that of H1 membranes, the histopathological analysis showed that the former exhibited most severe degradation was accompanied by a higher number of MNGCs at 10 days post-implantation. This suggests that the higher concentration of HMDI, while enhancing the collagenase resistance of the collagen membranes, induced higher quantity of multinucleated phagocytes, consequently resulting in faster cellular degradation. These results underscore the significant role of phagocytes, particularly macrophages and MNGCs of the pro-inflammatory phenotype, in material degradation. This degradation occurs through phagocytosis and the secretion of degrading mediators such as reactive oxygen species (ROS) and matrix metalloproteinases (MMPs), among others, to break down foreign objects (Sheikh et al., 2015). Consequently, mitigating inflammation post-implantation could theoretically facilitate optimal healing and extend the durability of collagen materials within the body.

However, complicating this model is the fact that PC membranes seem to successfully induce a mild inflammatory response with low MNGC numbers similar to that of RF membranes due to the incorporation of anti-inflammatory proanthocyanidins, do not demonstrate a longer standing time. This cellular response was combined with a complete fragmentation and a disintegration of the membrane into very small subunits, so that membrane functionality can no longer be assumed by day 30 post-implantation at the latest. Combined with the aforementioned mechanical property findings, this phenomenon may be attributed to the higher ductility of PC membranes. This characteristic renders PC membranes more prone to plastic deformation *in vivo* due to pressure, tensile, and shear forces exerted by the surrounding microenvironment. As a result, the increased surface area might make the membrane more vulnerable to fragmentation by cellular and enzymatic degradation. Altogether, only the EC-crosslinked membranes might be most suitable to offer barrier functionality up to day 90 post-implantation due to their special “fibrous” tissue integration pattern, but this is also very doubtful.

Macrophages also have been recognized as key regulators of vascularization, with distinct roles attributed to M1 and M2 phenotypes (Wang et al., 2021). It is widely accepted that M1 macrophages kickstart vascular regeneration by releasing vascular sprouting-associated factors, such as VEGF (Sunderkötter et al., 1991; Wang et al., 2021). Conversely, M2 macrophages play a crucial role in vascular maturation by recruiting pericytes and mesenchymal stem cells (MCS) through PDGF-BB secretion, both of which are crucial for vascular stabilization (Sunderkötter et al., 1991; Spiller et al., 2014). Thus, the precise timing and sequential activation of M1 and M2 macrophages are imperative for optimal vascularization outcomes. In this study, the GA, EC, and H1 membranes exhibited significantly lower levels of both vessel density and area fraction than the RF group at 10 days post-implantation, possibly due to their higher induction of M1 macrophages. Several studies have shown that increased population of M2 macrophages or enhanced M1 to M2 transition correlate with improved vascularization (Moore et al., 2018; Yin et al., 2020; Yang et al., 2023). Moreover, given the intricate interactions between M1 and M2 macrophages in vascularization, an increasing number of studies have shifted their focus to the M2/M1 ratio rather than the absolute population of macrophage subtypes. This notion is supported here by the findings in the PC group, where despite exhibiting M1 and M2 levels similar to those of RF, the lower M2/M1 ratio and significantly less vascularization compared to the RF group were observed.

Interestingly, the H2 group also induced a predominantly M1 macrophage response at this time point, yet it demonstrated vessel density and area fraction closest to RF. This could be attributed to their lower M1 levels and premature membrane collapse similar to that observed in RF, promoting premature growth of vascular-rich connective tissue into the membrane. Additionally, induced MNGCs also have the potential to secrete vascular endothelial growth factor (VEGF), thus contributing to the vascularization process (Barbeck et al., 2015; Al-Maawi et al., 2018). However, this vascularization advantage was not sustained. At 30 days post-implantation, the H2 group exhibited significantly lower vessel density than RF, possibly due to ongoing molecular crosstalk between multinucleated giant cells and macrophages. Numerous studies have demonstrated that vascularization is a complex process regulated by various inflammatory cells. For instance, Spiller et al. discovered that transient presence of M1 macrophages on the first day promoted blood vessel formation, while prolonged exposure on the third day led to vessel regression (Graney et al., 2020). The above findings underscore the intricate interactions among inflammatory cells, particularly macrophage subtypes, during vascularization.

When transferring the above results to clinical scenarios, it’s crucial to take the structural integrity of the membrane into account. On one hand, the fragmentation of the barrier membrane directly results in the loss of its barrier function, leading to the failure of Guided Bone Regeneration (GBR). On the other hand, premature fragmentation of the barrier membrane also expedites its degradation *in vivo*. Of the cross-linked membranes tested in this study, the EC membranes were stable materials within 30 days of implantation. Conversely, the other three membranes fragmented earlier, rendering them less suitable for application as Guided Bone Regeneration (GBR) barrier membranes. Furthermore, this study highlighted disparities between *in vitro* and *in vivo* investigations. Despite being chosen as a positive control for *in vivo* experiments due to cellular incompatibility, the GA group did not provoke adverse inflammatory reactions or fibrous capsule formation in the subcutaneous implant model. The restriction of the study lies in the subcutaneous model, which only reveals the biocompatibility and tissue response of the collagen membranes. A further study should be conducted using a bony implantation model which provides more relevant data in the context of bone tissue regeneration. However, this approach is complicated and often requires large animal testing.

## 5 Conclusion

The present study elucidates the material characteristics and distinct cell and tissue reactions elicited by collagen membranes cross-linked with various agents. Compared to the RF group, all cross-linking agents improved the enzyme resistance and standing time of the collagen membrane *in vivo*. In a subcutaneous implantation model, mononuclear cell responses were solely triggered in the RF group, whereas numerous multinucleated giant cells (MNGC) were observed in all cross-linked groups except for the PC group. With the emergence of abundant MNGC and M1 macrophages, membrane integrity deteriorated over the course of the experiment, leading to fragmentation and collapse. Among these, EC membranes remained structurally stable for up to 30 days, similar to GA group (negative control), whereas the other groups experienced structural breakdown earlier. Proanthocyanidins demonstrated anti-inflammatory properties, effectively mitigating foreign body reactions post-implantation. The PC group exhibited significantly reduced MNGC and M1 macrophages compared to other cross-linked groups, approaching levels seen in the RF group. However, it still faced premature membrane collapse *in vivo*, probably due to susceptibility to plastic deformation. Moreover, as the collagen membrane fragmented, transmembrane vascularization was observed across all tested groups, with histomorphometric findings highlighting intricate interactions of inflammatory cells during vascularization. Altogether, the EC membranes were stable materials within 30 days of implantation making them the most preferable material candidate out of the cross-linked membranes tested in this study. Conversely, the other three membranes fragmented earlier, rendering them less suitable for application as Guided Bone Regeneration (GBR) barrier membranes.

## Data Availability

The original contributions presented in the study are included in the article/supplementary material, further inquiries can be directed to the corresponding author.
